# An adapted smoking-cessation intervention for Turkish-speaking migrants in Switzerland: Predictors of smoking outcomes at one-year follow-up

**DOI:** 10.1371/journal.pone.0247157

**Published:** 2021-03-18

**Authors:** Raquel Paz Castro, Michael P. Schaub, Corina Salis Gross

**Affiliations:** Swiss Research Institute for Public Health and Addiction at the University of Zurich, Zurich, Switzerland; Universidade Federal de Sao Paulo, BRAZIL

## Abstract

**Background:**

Migrant populations usually report higher smoking rates. Among those migrant populations, Turkish- and Kurdish-speaking migrants are often overrepresented. Providing equal access to health services is one of the major challenges of our time. The need for adapted smoking-cessation treatments for Turkish-speaking populations to achieve equity in health led, in 2006, to the development and implementation of the *Tiryaki-Kukla* smoking-cessation program. The aims of the current study were to evaluate one-year quit rates for smoking-cessation courses held from 2006–2018 and investigate whether certain characteristics predict long-term smoking cessation or reduction.

**Methods:**

Program evaluation included a pre/post questionnaire (session 1/ 3 months after the quit day) and a follow-up telephone call twelve months after the quit day. To elucidate factors associated with long-term smoking cessation and reduction, Cox regression analysis and Weighted Generalized Equation Models were used.

**Results:**

Of the 478 who participated in smoking-cessation courses, 45.4% declared themselves non-smokers at one-year follow-up. This quit rate is higher than that achieved during the preliminary evaluation of the program involving 61 participants (37.7%). Predictors of long-term smoking cessation were course length (eight vs. six sessions) (95% CI = 1.04–1.36, *p* = .01), adherence to the course (95% CI = 0.98–0.99, *p*<0.01), use of pharmacotherapy or nicotine replacement therapy products (95% CI = 0.74–0.98, *p* = .02), and time passed in the morning until the first cigarette is smoked (95% CI^5min^ = 1.17–1.77, *p*<0.001; 95% CI^30min^ = 1.09–1.65, *p*<0.01). Predictors of change in cigarettes smoked per day among smokers were—the time passed until the first cigarette in the morning (5min *p* < .001; 30min *p* < .001; 60min *p* < .01)-, gender (*p* < .001), and level of motivation to quit at baseline (*p* = .04).

**Conclusions:**

Our findings are consistent with existing evidence supporting adapted smoking cessation interventions to reduce health inequity in migrant populations. However, achieving harm reduction in smokers with higher dependence scores remains challenging.

## Introduction

Smoking is a leading cause of disease [1]. Migrant populations usually report higher smoking rates, as evident in certain European countries [2–4] like Switzerland [5]. Among such migrant populations, Turkish- and Kurdish-speaking migrants are often overrepresented in various countries [3, 6, 7]. In Switzerland, daily smoking prevalence rates among Turkish-speaking men and women are roughly 55.2% and 29.5%, whereas they vary between 21.7% and 19.5%, among men and women, respectively, in the Swiss general population [7]. This can mainly be explained by the higher probability of migrants suffering from one of the so-called “social determinants of health” (e.g., poorer socio-economic status, being unmarried or cohabiting, unemployment, lower education level, language barriers) [3, 7, 8]. Providing equal access to healthcare services and reducing inequity in health is one of the major challenges of our time [9, 10]. In Switzerland, a comprehensive analysis of the range of services offered in the addiction area showed that people with a migration background, persons who are socially poorly integrated, and prison inmates are reached insufficiently by addiction treatments [11]. The goal of providing equal access to healthcare was added to the priorities of the Federal Office of Public Health (FOPH) in their released strategy paper “Health 2020” [12]. To achieve equal access to health services, barriers to those services must be sorted out. For example, mass media campaigns, mobile phone-based interventions, nicotine replacement therapy (NRT), and behavioral therapies in groups or single sessions have been found to be effective for smoking cessation [13–17]. Due to barriers like insufficient knowledge about healthcare services, language difficulties, and time constraints, migrant populations in Switzerland often fail to access regular smoking cessation treatments, like therapies offered by several providers (e.g., Cancer League, Lung League) [11, 18, 19]. It has also been shown that, in Austria, Turkish-speaking migrants participate less often in counselling programs than Austrian smokers, but are more willing to quit and have more previous cessation trials than Austrian smokers [20].

The need for adapted smoking cessation treatments for Turkish-speaking populations to achieve equity in health led, in 2006, to the development and implementation of the Tiryaki-Kukla program (www.tiryakikukla.ch) [18]. In short, the Tiryaki-Kukla program includes informative talks and smoking cessation courses that are grounded in behavioral therapy. It aims to alter the behavior of its target group (smoking cessation or reducing the number of cigarettes smoked) and to protect them from passive smoking in clubs and groups. Since 2010, the adapted smoking cessation program has been integrated into the National Tobacco Prevention Program. Over the entire implementation period, from 2006 to 2019, 6’605 Turkish-speaking migrants in Switzerland received tobacco-related information and were offered tailored treatment for smokers and smoking relatives. From these contacts, 81 smoking-cessation courses have resulted, to date. The main aims of the current study were (1) to analyze one-year quit rates for the courses held from 2006 to 2018; and (2) to elucidate factors associated with smoking cessation and reduction.

## Materials and methods

### Design, subject recruitment, and inclusion/exclusion criteria

This study is a longitudinal field study with a one-group pre-post follow-up design. Predictors associated with long-term smoking cessation and reduction were explored. Data were collected anonymously and matching of baseline, 3-month and one-year data was ensured by key codes, which were kept securely by the principal investigator of the study. The collection of data was reviewed and approved by the Tobacco Control Fund from the Federal Office of Public Health of Switzerland. The study is not a clinical trial; it is a self-evaluation of a program which is part of Swiss Public Health. This is why the Swiss Human Research Act (HRA) of 2014, regulating research on human participants, did not apply. The HRA regulates which projects are considered clinical research and, thus must undergo a review process by a corresponding ethics committee in Switzerland.

From March 2006 to June 2018, a multi-modal strategy of subject recruitment was pursued to ensure a diverse sample of Turkish-speaking migrants. The typical procedure was to initially hold an informative talk in Turkish at clubhouses or organizations of the Diaspora in Switzerland to inform all interested persons about the hazards of smoking. With these talks, not only smokers, but also non-smokers possibly affected by the smoking behavior of a relative were reached. A total of 137 talks were held between 2006 and 2018, reaching approximately 5’744 persons. The aim of these talks was to indirectly recruit or influence smokers over their non-smoking relatives. After the talks, key members of the community or informal groups helped the coaches to recruit smokers interested in a cessation course.

Subjects were also personally recruited at a variety of events within the Turkish-speaking community in Switzerland (e.g., clubhouses, mosques) with culturally-sensitive posters and flyers. Further, personal networks of the smoking cessation coaches were used to reach smokers and form cessation courses. Additionally, advertisements were distributed via different media (internet and print), local radio stations, national Turkish television and, most recently, Facebook. After 2016, greater dispersion of advertisements was pursued during Ramadan, based on the assumption that smokers’ motivation for quitting would be greatest during this period. From 2016 to 2018, 65 mosques were visited during Ramadan to inform mosque attendees about smoking hazards and recruit persons interested in a smoking cessation course.

The inclusion criterion for participation in a cessation course was smoking cigarettes at any level and being at least 18 years old. Sufficient or insufficient mastery of the language of the host country was not an inclusion/exclusion criterion. A total of 71 courses encompassing 478 participants were formed. All subjects smoked cigarettes at least monthly and were, thus, eligible for the study. Eligible persons were informed about the study purpose, that they could cancel their participation at any time without negative consequences, and that all of their data would be treated confidentially. All 478 course participants agreed to take part in the study.

### Smoking cessation courses

Development of the smoking cessation program for Turkish-speaking migrants in Switzerland has been described elsewhere [18]. In short, these smoking cessation courses are grounded in behavioral therapy and were adapted from the weekly group-counseling sessions applied by Cancer League Zurich. The adapted course material was double-checked by key members of the Turkish and Kurdish communities living in Switzerland, for language, cultural and health literacy issues. Health literacy was defined as “the degree to which individuals have the capacity to obtain, process, and understand basic health information and services needed to make appropriate health decisions” [21]. Three types of health literacy are known: basic/functional health literacy, communicative/interactive health literacy, and critical health literacy [22]. Beyond translation of the course material, adaptations made to improve health literacy—more precisely, functional health literacy (reading and writing)—were a) replacing symbolic references, like Swiss food examples with typical Turkish food, and b) enriching the manual with more visual elements to accommodate lower levels of education. Interactive health literacy (installing favorable social situations) was fostered by looking at typical social situations for Turkish-speaking migrants to enhance relapse prevention; and discussing attractive activities that could prevent weight gain that might result from smoking cessation. Critical health literacy (analyzing information and current practices in healthcare systems) was targeted by discussing different health information websites or advantages and disadvantages of NRT products. At every weekly group session, the level of carbon monoxide (CO) in the breath of every participant was analyzed with a piCO smokerlyzer (Bedfont Scientific Ltd.) and documented by the coach.

One female and one male applicant with a Turkish background were recruited in 2006 and received intensive training as smoking cessation coaches. The reason for this was to be able to offer women’s only and men’s only groups in the Turkish language. The male coach had to be replaced in 2016.

**Fig 1:**
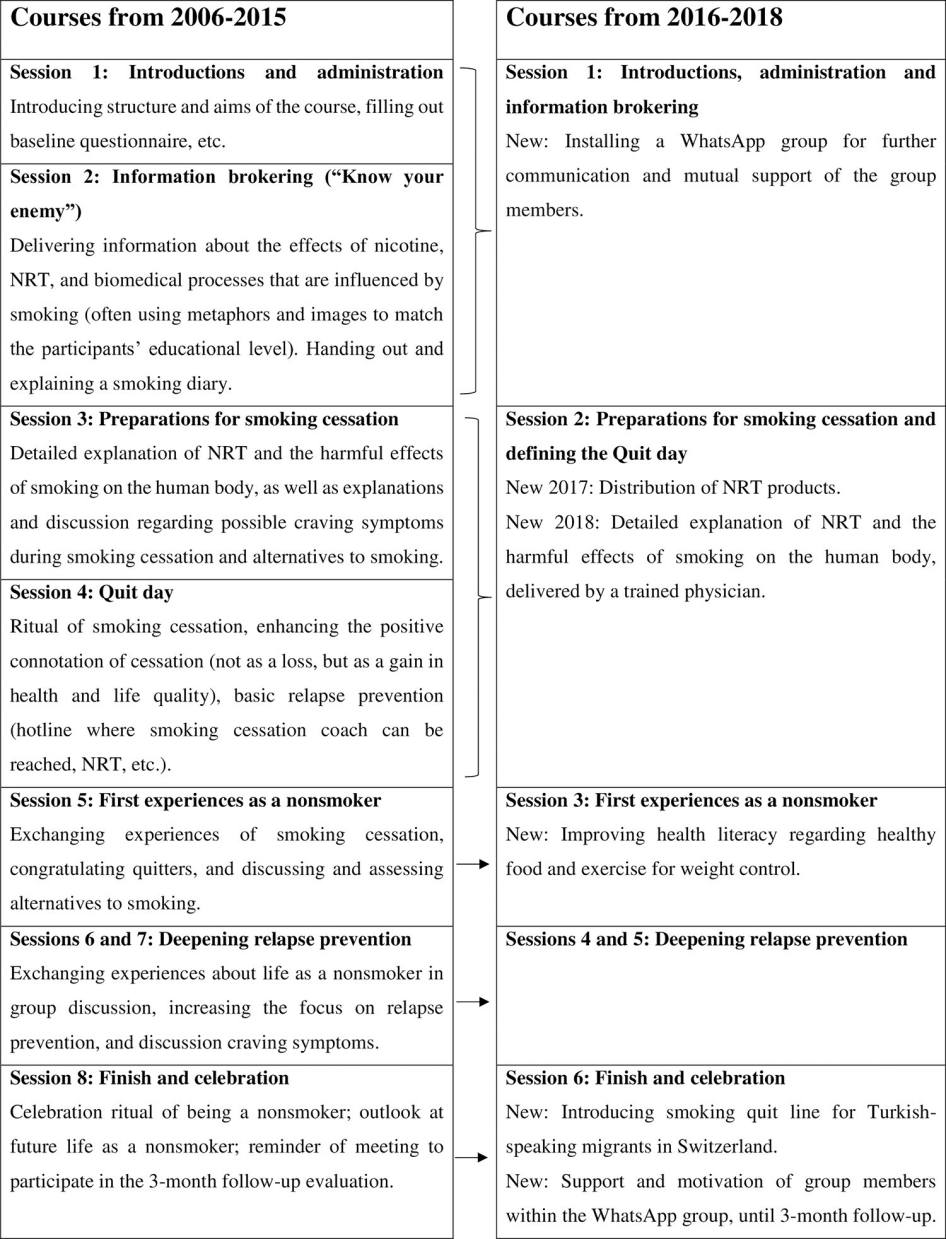
Structure of the weekly group-counseling sessions before and after 2016.

Over the duration of this study, the smoking cessation courses were altered several times. From 2006–2010, the smoking cessation courses consisted of eight weekly counseling sessions for groups and four single counseling sessions. From 2010–2015, only the eight weekly counseling sessions for groups were retained, since single counseling sessions were found to be unattractive to participants [18]. Participants who needed more prolonged counseling were then forwarded to the National Quit Smoking Helpline, which offers comprehensive counseling in Turkish. Quit day was usually held collectively during the fourth group session. From 2016 onwards, an adaptation to six weekly counseling sessions for groups was performed, conforming to the procedures of Cancer League Zurich, which also condensed its course manual. Quit day was then held collectively between the second and the third group session. Also in 2016, the Turkish course material was adapted to Albanian and this new translation integrated into the National Tobacco Prevention Program; this, however, is not part of the current study and will be discussed further in a future paper. From 2017 onward, NRT products—like nicotine gums, nicotine patches, and nicotine inhalers—were distributed, at no charge, to course participants who were willing to use them for quitting. From 2018 onward, the second group session was enriched by including a talk by a trained physician on the hazards of smoking. Both a male and female physician were recruited for this purpose. Fig 1 compares the structure of the group sessions before and after 2016.

### Measures and outcome criteria

Effects of the weekly group-counseling sessions were assessed with several instruments, including a pre/post questionnaire (session 1/ 3 months after the quit day) and a follow-up telephone call twelve months after the quit day. The pre and post questionnaires were pre-tested on five Turkish-speaking migrants with different levels of education and health literacy, and different genders using the “think aloud” method of “the cognitive interview” [23]. After the first smoking cessation course, the questionnaires were re-adapted to enhance their comprehensibility. From 2006 to 2018, the questionnaires were validated annually, with coaches reporting any difficulties participants appeared to have while completing the assessments. In 2015, the questionnaire was completely revised, inviting four Turkish-speaking migrants with different backgrounds to participate again in a “cognitive interview”, leading to the actual questionnaires (S1–S6 Files).

The baseline questionnaire was filled out by participants of the course as a paper-pencil version within the first course session. Both the three-month and one-year follow-up assessments were conducted via a telephone call by trained interviewers who were unknown to the participants and spoke their mother tongue.

Core measures included in all the pre/post questionnaires were socio-demographic and tobacco-related variables. *Socio-demographic core variables* were gender, age, marital status, parental status, mother tongue, Swiss nationality, level of educational attainment, employment status, age of first contact with smoking, and age of onset of regular smoking. *Tobacco-related core variables* were smoking status, number of cigarettes smoked per day (CPD), intention to quit smoking, number of quit attempts, duration of the longest prior quit attempt, use of any pharmacotherapy or NRT, whether or not they lived in a smoke-free home, whether or not smoking was reduced among peers, whether or not they attended tobacco-related activities at a mosque/club, and cigarette dependence. Intention to quit smoking was assessed asking participants how strong their readiness to quit smoking was at that time, ranging from 0 ‘not ready at all’ to 10 ‘I am very ready’. Following the recommendation of Baker et al. [24], the level of cigarette dependence was rated using the first item of the Fagerström test [25]: “How soon after waking do you smoke your first cigarette?” (available response options: within 5 minutes; within 5–30 minutes, within 31–60 minutes; after 60 minutes).

Follow-up calls one year after the quit day focused on smoking status (“not having smoked a puff” within the past 30 days, according to criteria published by the Society for Research on Nicotine and Tobacco [26]). After 2014, the number of cigarettes smoked per day (CPD) was also assessed at one-year follow-up. Smoking status and CPD were our study’s primary outcomes. Questionnaires for non-smokers were shorter and skipped questions related to actual smoking. For an overview of measurements and instruments, see Table 1.

**Table 1:** Overview of measurements and instruments.

Assessments / instruments	Baseline	3-month follow-up, smokers	3-month follow-up, non-smokers	1-year follow-up, smokers	1-year follow-up, non-smokers
**Socio-demographics**	x				
***Primary outcomes***					
**Smoking status** ^**1**^	x	x	x	x	x
**Cigarettes smoked per day (CPD)** ^**1**^^,^^**2**^	x	x		x	
***Secondary outcomes***					
**Quit attempt**	x	x			
**Quit attempt duration**		x			
**Use of pharmacotherapy / NRT**		x	x		
**Smoke-free home** ^**3**^	x	x	x		
**Smoking reduced in own peers** ^**2**^		x	x		
**Continuing tobacco-related activities at a mosque/ club** ^**2**^		x	x		

^1^ 30 days before assessment.

^2^ Question included after 2014.

^3^ Question included after 2010.

NRT = nicotine replacement therapy.

### Data analysis

First, we checked how participants lost to follow-up after the baseline assessment differed from those seen at the three-month and one-year follow-up evaluations. We analyzed differences in categorical variables by Pearson chi-square tests, and differences in continuous variables by unpaired Student’s *t* tests.

The primary outcomes—smoking status and cigarettes smoked per day—were examined as follows: To analyze (1) longitudinal changes and (2) significant predictors of change in cigarettes smoked per day in smokers, we used Weighted Generalized Estimating Equation (WGEE) analysis. WGEE is a repeated-measures regression model that takes into account the correlation of repeated measures within each subject [27]. WGEE makes minimal assumptions about time dependence and uses all available data, irrespective of single missing values. WGEE is applied when the assumption of missing completely at random (MCAR) is violated. In WGEE, attrition bias is minimized through the estimation of weights. We estimated weights, as suggested by Salazar and colleagues [28], to control for attrition bias at the three-month and one-year follow-up assessments.

In round 1 of analysis, we only included the time variable within the WGEE models to examine for significant changes in primary outcomes over the study’s course. In round 2, the following baseline variables were added: gender, age, marital status, children in own household, level of educational attainment, mother tongue, age of smoking onset, expectancy of course success, participation rate, use of pharmacotherapy or NRT, motivation for quitting, number of smoking persons in household, and number of smoking friends. Through a hierarchical, backward procedure, whereby we removed predictors with the highest p value one at a time, we retained the significant predictors of change in frequency of smoking within the model.

To analyze the effects of the referred baseline variables as predictors of smoking status, we used Cox regression analysis. Similar to WGEE analysis, predictors with the highest p values were removed, one at a time, until only significant predictors were retained in the final Cox regression model.

For both our WGEE and Cox regression analysis, we controlled for the year of course attendance (before January 1, 2016 vs. January 1, 2016 onwards), since course duration changed from eight to six sessions from 2016 onwards. An alpha level of 0.05 (2-tailed) was chosen for all statistical tests conducted in the study.

Descriptive statistics were used to analyze changes in secondary outcomes. All analyses were performed using the statistical tools SPSS version 22 and R version 3.6 via the geepack [29] and survival [30] packages.

## Results

### Participants’ baseline characteristics

All persons who attended the smoking cessation courses were eligible for the study and answered the pre-questionnaire. Baseline characteristics of the study sample are summarized in Table 2. The majority of participants indicated high levels of nicotine dependence (81.8% were daily smokers and 64.2% smoked their first cigarette in the morning within 5 to 30 minutes of awakening). Some participants were characterized by high psychosocial vulnerability, like being divorced/separated or widowed (18.2%) or being unemployed (31.6%).

**Table 2:** Baseline characteristics of the study sample (n = 478).

Variable		
**Sex (%)**	male	226 (47.3)
	female	249 (52.1)
	missing	3 (0.6)
**Age, *M (SD)*** ^**a**^		42.7 (10.3)
**Marital status (%)**	single	71 (14.9)
	married / stable partnership	306 (64.0)
	married and living apart	9 (1.9)
	divorced / separated	75 (15.7)
	widowed	12 (2.5)
	missing	5 (1.0)
**Children living in same household (%)**	no	185 (38.7)
	yes	289 (60.5)
	missing	4 (0.8)
**Mother tongue (%)**	Turkish	319 (66.7)
	Kurdish	149 (31.2)
	other	8 (1.7)
	missing	2 (0.4)
**Living area (%)**	urban	347 (72.6)
	rural	131 (27.4)
**Swiss nationality (%)** ^**b**^	no	295 (61.7)
	yes	126 (26.4)
	missing	57 (11.9)
**Highest education level (%)**	no school attended	22 (4.6)
	primary school (years 7–12)	81 (16.9)
	middle school (years 12–15)	126 (26.4)
	upper school (years 15–18)	188 (39.3)
	university (years 18+)	52 (10.9)
	missing	9 (1.9)
**Working status (%)**	yes, full-time	141 (29.5)
	yes, part-time	74 (15.5)
	housewife	63 (13.2)
	in education	11 (2.3)
	not working or in school	151 (31.6)
	missing / no comment	38 (7.9)
**Tobacco smoking status (%)** ^**b**^	daily smoker	391 (81.8)
	occasional smoker	20 (5.2)
	missing	62 (13.0)
**Number of cigarettes smoked per day (CPD), *M (SD)*** ^**c**^		17.9 (9.5)
**Fagerström, (%)**	5 min	150 (31.4)
	6–30 min	157 (32.8)
	31–60 min	83 (17.4)
	60+ min	80 (16.7)
	missing	8 (1.7)
**First session CO, *M* (range, *SD*)** ^**d**^		20.8 (1–64, 10.3)
**Intention to quit smoking (0–10), *M (SD)*** ^**e**^		4.8 (2.5)
**Previous quit attempts (%)**	no	117 (24.5)
	yes	355 (74.3)
	missing	6 (1.3)
**Number of previous quit attempts, *M* (range, *SD*)** ^**f**^		2.3 (0–20, 2.3)
**Age of first contact with smoking, *M (SD)*** ^**g**^		17.2 (4.6)
**Age of onset of regular smoking, *M (SD)***^**h**^		20.0 (5.3)

^a^ missing information n = 21.

^b^ question included after 2010.

^c^ missing information n = 8.

^d^ missing information n = 19.

^e^ missing information n = 10.

^f^ missing information n = 27.

^g^ missing information n = 12.

^h^ missing information n = 28.

### Course retention and satisfaction

Over the study period of twelve years, a mean course retention rate of 93.4% (SD: 16.1%) was achieved. Only a few participants (27/478, 5.6%) discontinued treatment and dropped out of the course. In total, only 22 (4.6%) participants failed to complete the three-month or one-year follow-up assessments. Reasons for non-participation at three-month follow-up were 1) no response (n = 20) and 2) incorrect number (n = 2), while reasons for non-participation at one-year follow-up were 1) no response (n = 19), 2) incorrect number (n = 2), and 3) refusal (n = 1). At three-month follow-up, participants were asked if the course was helpful for quitting smoking. Out of 374 participants, 240 (64.2%) indicated that the course was very helpful and 73 (19.5%) that it was helpful. Thirty-two (8.6%) participants responded that they could not tell and 29 (7.7%) that the course was not helpful (at all). Participants were also asked if the course had been helpful in other areas, such as everyday questions, networking, etc. Almost all participants (374/425, 88.0%) stated that it had been (very) helpful in other areas. Only 25/425 (5.9%) indicated the opposite. Lastly, they were asked if they would recommend this course to other peers. The vast majority (414/424, 97.6%) indicated that they (certainly) would recommend the course to others, four (0.9%) could not tell, and six (1.4%) said they would not recommend it.

### Attrition

Attrition analysis revealed that 3-month assessments were more likely to be completed by participants who were older when they started to smoke on a regular base (*t* = 1.97, *df* = 448, *p* = .05) or who had attended a higher number of course sessions (*t* = 2.94, *df* = 20.3, *p* = .008). The one-year assessments were more likely to be completed by Turkish-speaking than Kurdish-speaking migrants (*X*^*2*^ = 20.93, *df* = 3, *p* < .001).

### Changes in tobacco-related outcomes

Table 3 summarizes changes in the primary and secondary outcomes. Positive changes were observed in all domains. The CO measurement in the last session of the course revealed that 70.3% of the participants could be considered non-smokers (defined as a CO score < 8 parts per million (Ppm)). Noteworthy is that 50.2% of the participants declared themselves non-smokers at three-month follow-up and 45.4% still defined themselves as non-smokers after one year. More than two thirds of the non-smokers at T1 (153, 71.2%) indicated that their house was smoke free, a percentage that was more than double that at baseline (73, 34.0%).

**Table 3:** Number of participants and changes in tobacco-related outcomes between baseline, 3-month and 1-year follow-up.

		Baseline (T0)	3 months (T1)	1 year (T2)
**Primary outcomes**				
** Smoking status (%) (N = 478)**	smoker	478 (100)	216 (45.2)	239 (50.0)
	non-smoker	-	240 (50.2)	217 (45.4)
	missing	-	22 (4.6)	22 (4.6)
** Cigarettes per day, *M (SD)***	smokers only, before 2014 (n = 203)	20.4 (9.8)	14.5 (8.6)	-
	smokers only, after 2014 (n = 95)	19.2 (9.7)	11.9 (8.6)	6.8 (6.6)
**Secondary outcomes**				
** Quit attempts before T1, smokers only (%) (n = 216)**	no		130 (60.2)	-
	yes		81 (35.7)	-
	missing		10 (2.3)	-
** Quit attempt duration, *M (SD)*, (n = 76)**			23.6 (28.4)	-
** Use of any pharmacotherapy / NRT before T1 (%)**	all participants (N = 478)		158 (33.1)	-
	smokers at T1 (n = 216)		62 (28.7)	-
	non-smokers at T1 (n = 240)		96 (40.0)	-
** Smoke-free home (%)** ^**a**^	all participants (n = 422)	136 (32.2)	222 (52.6)	-
	smokers at T1 (n = 185)	58 (31.4)	69 (37.3)	-
	non-smokers at T1 (n = 215)	73 (34.0)	153 (71.2)	-
**Smoking reduced in own peers** ^**b**^ **(%) (n = 144)**	yes	-	6 (4.2)	-
	no	-	114 (79.2)	-
	missing	-	24 (16.7)	-
**Continuing tobacco-related activities in mosque/ club** ^**b**^ **(%) (n = 137)**	yes	-	66 (83.3)	-
	no	-	28 (6.6)	-
	missing	-	43 (10.1)	-

^a^ Question included after 2010.

^b^ Question included after 2014.

NRT = nicotine replacement therapy

Among the participants who still smoked at T1, 35.7% had seriously tried to quit smoking at least once, and the number of completely smoke-free houses increased from 31.4% to 37.3%. Smokers over the study period significantly reduced the quantity of cigarettes they smoked per day (WGEE: *β*0^Intercept^ = 18.79, *β*1^3months^ = -3.21, SE = 0.57, *p* < .001, *β*2^1year^ = -10.64, SE = 0.75, *p* < .001).

### Predictors of change in primary outcomes

**Fig 2:**
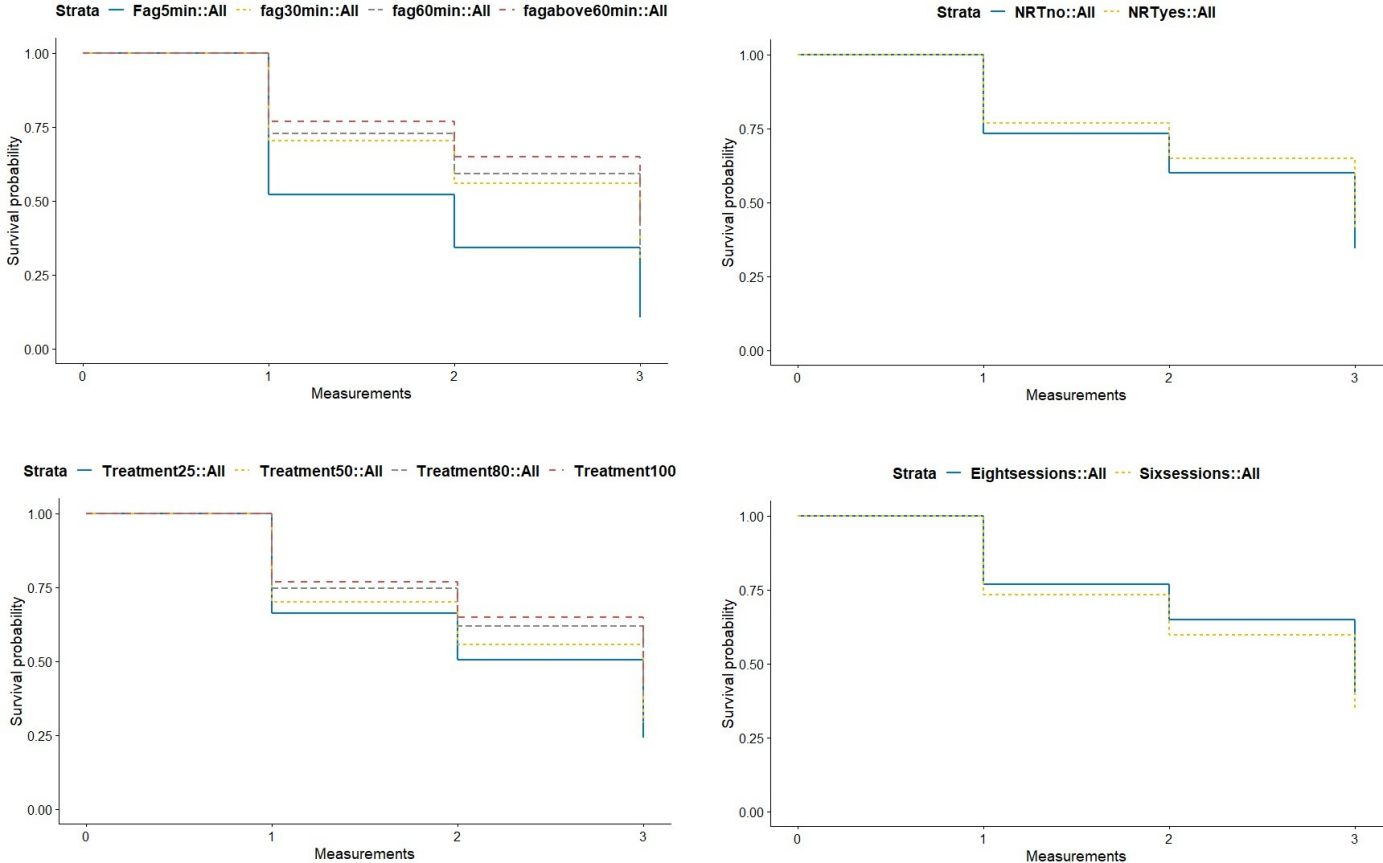
Survival courves for staying non-smoker for the comparisons of 1) different grades of nicotine dependence based on the first item of the Fagerstrom test, 2) use of NRT, 3) different participation rates, and 4) different length of the course.

Cox regression analysis revealed that the main predictors of smoking at follow-up were attendance at six versus eight course sessions, use of pharmacotherapy or NRT, participation rate, and the time that passed after awakening in the morning before they smoke their first cigarette. Participants of the shorter courses had a higher risk of being a smoker at follow-up (Odds ratio (OR) = 1.18, CI 95% = 1.04–1.36, *p* = .01). Consistent with this, attending a greater number of sessions resulted in a lower risk of being a smoker at follow-up (OR = 0.99, CI 95% = 0.98–0.99, *p*<0.01). The use of pharmacotherapy or NRT also reduced the risk of being a smoker at follow-up (OR = 0.85, CI 95% = 0.74–0.98, *p* = .02). Lastly, compared to participants who smoked their first cigarette more than one hour after awakening in the morning, participants who smoked their first cigarette within the first 5 minutes or within the first half hour had a higher risk of still being a smoker at follow-up (OR^5min^ = 1.44, CI 95% = 1.17–1.77, *p*<0.001; OR^30min^ = 1.34, CI 95% = 1.09–1.65, *p*<0.01). No differences were found between participants who smoked their first cigarette between 30 and 60 minutes and more than 60 minutes after awakening. In Fig 2, diverse survival curves are displayed comparing pharmacotherapy use, course attendance, participation rates and nicotine dependency.

WGEE models revealed the following significant predictors (besides time) of change in cigarettes smoked per day among smokers: gender, motivation to quit at baseline, and time passed in the morning until the first cigarette was smoked. Female smokers and more-motivated smokers reduced their daily smoking more pronouncedly than males (*β*1 = -4.67, SE = 0.63, *p* < .001) and less-motivated (*β*1 = -0.19, SE = 0.09, *p* = .04) smokers did, respectively. Lastly, relative to participants who smoked their first cigarette more than one hour after awakening in the morning, participants who smoked their first cigarette within the first five minutes (*β*1 = 10.65, SE = 0.95, *p* < .001), within the first half hour (*β*1 = 6.39, SE = 0.88, *p* < .001) or within the first hour (*β*1 = 2.61, SE = 0.99, *p* < .01) reduced their daily cigarette consumption to a lesser degree.

## Discussion

### Principle findings

Approximately fifty percent of the Turkish-speaking migrants who participated in adapted group courses in Switzerland reported being smoke free at one year follow-up. This is even a higher rate than was observed in the preliminary evaluation of the program by Schnoz and colleagues (37.7%) [18] and higher than the quit rate for a comparable smoking cessation program in Germany (31.8%) [31]. Noteworthy is that, contrary to the first evaluation, the courses were held all over Switzerland and offered in urban as well as rural areas. In addition, the effect seemed to be resistant to changes in the program and staff.

Nonetheless, reducing the group counseling courses from eight to six might have reduced the chances for some smokers to reduce their smoking or quit altogether. On the other hand, delivering NRT products free of charge to participants increased the odds of smoking cessation success, something already observed in our previous evaluation [18]. Further analysis of the cost-effectiveness of this combination (fewer lessons/free NRT) is needed.

What becomes clear from our results is that the group counseling course and NRT products are still insufficient for highly-addicted or unmotivated individuals, in terms of both smoking cessation and reduction. Evaluation of the German smoking cessation program also identified the lowest quit rates in participants with higher dependence scores [31]. Our findings also are consistent with the latest Cochrane review [32], which detected only minimal evidence of NRT effectiveness fostering harm reduction in smokers who were unwilling to quit. Ways to achieve harm reduction in this subgroup–like electronic nicotine delivery systems (EC)—must be reconsidered. To date, research indicates little evidence supporting the efficacy of EC, but also few serious adverse events [33, 34]. While an ongoing study is about to replicate these findings on a larger scale in Switzerland [35], the acceptance and usability of such devices among migrants would still need to be determined and could be a next step of testing within adapted group counseling courses.

### Strengths and limitations

Strengths of this study include 1) the evaluation of courses over a decade, 2) our validation of smoking status during the group counseling sessions by measuring exhaled CO, and 3) the high follow-up rates relative to similar studies. The long time-span examined allows us to conclude that our results were quite robust to changes in recruitment, program, and staff; but these factors were not examined individually. For example, the effect of recruiting religious smokers during Ramadan on outcomes cannot be established and could be of interest for future research.

The high follow-up rates were probably achieved thanks to the relationship-led approach on which this adapted program relies. During the first and last course sessions, the coaches introduced to the participants the interviewer responsible for the telephone assessments. The interviewer, in turn, informed each participant at the beginning of the assessment that they were calling in the name of the respective coach. The research institution was only mentioned afterwards. This approach is likely feasible in other groups or countries, as long as reciprocal social relationships are used systematically throughout all phases of the project.

For the three-month and one-year follow-up assessments, we had to rely on self-reports, which is the most notable limitation of this study. Also, the study design allowed no causal inferences about the effect of the intervention on quit rates. Nonetheless, the quit rates we observed within this study are higher than the naturalistic quit rates observed among smokers within the Swiss general population (24.0%) [36], who mainly quit without taking advantage of any support line or NRT products.

## Conclusions

The findings of this study are further evidence supporting the effectiveness of adapted smoking cessation interventions among migrant populations [37]. Moreover, they demonstrate that using a proactive recruitment strategy and offering a course at no charge increases the chances of enrolling a diverse group of Turkish-speaking smokers. In so doing, this further increases the likelihood of health equity in this subgroup. However, it remains a challenge to achieve harm reduction in smokers with higher dependence scores. Testing electronic nicotine delivery devices should be considered for this subgroup, including examining their acceptance and usability, and long-term adverse effects

## Supporting information

S1 File İsviçre’de yaşayan Türkçe konuşan göçmenler için sigarayı bırakma kursu değerlendirme anketi. T1-Anket.
(PDF)

S2 File Evaluation of smoking cessation courses for Turkish-speaking migrants in Switzerland. T1 questionnaire.
(PDF)

S3 File İsviçre’de yaşayan Türkçe konuşan göçmenler için sigarayı bırakma kursu değerlendirme anketi. Sigara İçmeyenler İçin T2 anketi.
(PDF)

S4 File Evaluation of smoking cessation courses for Turkish-speaking migrants in Switzerland. T2 questionnaire for non-smokers.
(PDF)

S5 File İsviçre’de yaşayan Türkçe konuşan göçmenler için sigarayı bırakma kursu değerlendirme anketi. Sigara içenler için T2 Anketi.
(PDF)

S6 File Evaluation of smoking cessation courses for Turkish-speaking migrants in Switzerland. T2 questionnaire for smokers.
(PDF)
