# Supplementary material for: An adapted smoking-cessation intervention for Turkish-speaking migrants in Switzerland: Predictors of smoking outcomes at one-year follow-up
Source: PLoS One. 2021 Mar 18;16(3):e0247157. doi: 10.1371/journal.pone.0247157 (PMC7971503; doi:10.1371/journal.pone.0247157)
Supplement: S1 File — T1-Anket. (PDF) [file pone.0247157.s001.pdf]

Schweizer Institut für Sucht- und Gesundheitsforschung
Swiss Research Institute for Public Health and Addiction
Institut suisse de recherche sur la santé publique et les addictions

Kamu Sağlığı ve Bağımlılık Araştırma Enstitüsü

İsviçre'de Yaşayan Türkçe Konuşan Göçmenler İçin Sigarayı Bırakma Kursu Değerlendirme Anketi

T1-Anket

Lütfen **DOLDURMAYINIZ**. Bu bölüm **KURS YETKİLİLERİ** tarafından doldurulacaktır.

Probanden-Nummer:	|__ __ __ __ __ __ __ ____|	*pbnr*		
Datum des ersten Kursbesuchs:	__ __ : __ __ : __ __ __ __<br>TT MM JJJJ	*t1work_tag*	*t1work_mon*	*t1work_jahr*
Datum an dem FB T1 ausgefüllt wurde:<br>(falls nicht identisch mit Datum des ersten Kursbesuchs)	__ __ : __ __ : __ __ __ __<br>TT MM JJJJ	*t1fb_tag*	*t1fb_mon*	*t1fb_jahr*

Sigara İçme Davranışları ile İlgili Sorular

1. Ne kadar sıklıkta sigara içiyorsunuz?
Lütfen sadece bir kutu işaretleyiniz

- ☐ 1 Hergün *t1häufig*
- ☐ 2 Haftada birden fazla (haftada ___ kez) *t1häufig2*
- ☐ 3 Haftada bir kez *t1häufig3*
- ☐ 4 Daha az (ayda ___ kez) *t1häufig4*

2. a) İlk kez sigara içtiğinizde kaç yaşındaydınız?
______ yaşındaydım *t1ziga*

b) Düzenli olarak sigara içmeye başladığınızda kaç yaşınızdaydınız?
_______ yaşındaydım *t1zigb*

3. Sigara içtiğiniz bir günde, genel olarak kaç tane sigara içiyorsunuz?

a) Günde _______ tane sigara *t1freqzigi*
b) Günde _______ tane puro *t1freqziga*
c) Günde _______ kere pipo *t1freqpfei*
d) Günde ________ kere nargile *t1freqw*
e) Günde ________ tane "Joint" (Haşhaş / „Ot" / Esrar) *t1freqjo*
f) Günde ________ tane e-sigara *t1freqezig*

4. Normalde sabah kalktıktan ne kadar sonra ilk sigaranızı içiyorsunuz?
Sadece bir kutu işaretleyiniz.

- ☐ 4 5 dakika içinde *t1fag1*
- ☐ 3 6 - 30 dakika sonra
- ☐ 2 31 - 60 dakika sonra
- ☐ 1 60 dakikadan fazla

5. Evinizde nerelerde ve ne kadar sıklıkta sigara içiliyor?

Her satırda bir kutuyu işaretleyiniz.

	sık sık	nadiren	hiç	
a) Oturma odası	☐ 2	☐ 1	☐ 0	*t1homea*
b) Yatak odası	☐ 2	☐ 1	☐ 0	*t1homeb*
c) Çocuk odası	☐ 2	☐ 1	☐ 0	*t1homec*
d) Banyo / Tuvalet	☐ 2	☐ 1	☐ 0	*t1homed*
e) Mutfak	☐ 2	☐ 1	☐ 0	*t1homee*
f) Balkon / Bahçe / Teras	☐ 2	☐ 1	☐ 0	*t1homef*
g) Başka yer (yazınız):___________________________	☐ 2	☐ 1	☐ 0	*t1homeg* *t1homeg_off*

6. Arabada sigara içmek: Sizin veya birlikte yaşadığınız kişinin arabası var mı?

☐ 1 Hayır *t1autoa*

☐ 2 Evet

	sık sık	nadiren	hiç	
a) Eğer evetse, ne kadar sıklıkla bu arabada sigara içiliyor?	☐ 2	☐ 1	☐ 0	*t1autob*

7. Evinizde kaç kişi yaşıyorsunuz? (Siz dahil)

Lütfen sayısını yazınız: |______| kişi yaşıyoruz. *t1mitbewo*

8. Sizinle aynı evde yaşayanlardan kaçı sigara içiyor? (Siz dahil)

Lütfen sayısını yazınız: |______| kişi sigara içiyor *t1mitbewora*

9. Sizinle aynı evde yaşayan çocuğunuz var mı?

☐ 2 Evet *t1kind*

☐ 1 Hayır

a) Evetse, kaç çocuk sizinle birlikte yaşıyor |______| *t1anzkia*

b) Çocukların yaşları |______| |______| |______| |______| |______| *t1altki1-5*

1. Çocuk 2. Çocuk 3. Çocuk 4. Çocuk 5. Çocuk

10. İsviçre'deki aile ve arkadaşlarınızdan en yakınınızdaki 10 kişiden kaçı sigara içiyor?

Lütfen sayısını yazınız: |______| Kişi *t1personen*

11. Genel olarak Sigara İçme konusunda ne düşünüyorsunuz? Aşağıdaki söylemler sizce ne kadar doğru?

Lütfen her şıkta bir kutu işaretleyiniz. Burada doğru ya da yanlış cevap yoktur. Lütfen düşünceniz neyse onu işaretleyiniz.

		**Tamamen Katılıyorum**	**Biraz Katılıyorum**	**Pek Katılmıyorum**	**Hiç Katılmıyorum**	
		1	2	3	4	
a)	Sigara içmek can sıkıntısına iyi gelir..	☐	☐	☐	☐	*t1ea*
b)	Sigara ardında kötü bir koku bırakır	☐	☐	☐	☐	*t1eb*
c)	Sigara içmek modern bir görünüş sağlar	☐	☐	☐	☐	*t1ec*
d)	Sigara içmek cildi daha çabuk yaşlandırır	☐	☐	☐	☐	*t1ed*
e)	Sigara insanı gevşetir ve rahatlatır.	☐	☐	☐	☐	*t1ee*
f)	Sigara içmek diğer insanların sağlığını da bozar	☐	☐	☐	☐	*t1ef*
g)	Sigaranın tadı güzeldir	☐	☐	☐	☐	*t1eg*
h)	Restorant, kahve ve bar gibi kamuya açık yerlerde sigara içilmesinin yasaklanması konusunda ne düşünüyorsunuz?	☐	☐	☐	☐	*t1eh*

Sigarayı Bırakmakla İlgili Sorular

12. Sigara içme kursundan önce hiç sigarayı bırakmayı denediniz mi?

☐ 1 Hayır *t1stop*

☐ 2 Evet

Evetse, kaç kere? _____ kere *t1anzstop*

13. Kendinizi sigarayı bırakmak için ne kadar hazır hissediyorsunuz?

Lütfen tahmininize en yakın sayıyı işaretleyiniz:

t1stoptermo

0 —— 1—— 2 —— 3 —— 4 —— 5 —— 6 —— 7 —— 8 —— 9 —— 10

0 = hiç hazır değilim 10 = tamamen hazırım

14. Bu kursun da yardımıyla bir sene sonra hala sigara içmeyen bir kişi olacağınıza inancınız ne kadar?

Lütfen tahmininize en yakın sayıyı işaretleyiniz:

t1erwartung

0 —— 1—— 2 —— 3 —— 4 —— 5 —— 6 —— 7 —— 8 —— 9 —— 10

0 = hiç 10 = tamamen

Kişisel Sorular

15. Cinsiyetiniz?

☐ 1 Erkek *t1sex*

☐ 2 Kadın

16. Kaç yaşındasınız? ***t1alter***

___________ yaşındayım

17. Şu anki medeni durumunuz?

Lütfen yalnızca bir kutu işaretleyiniz.

☐ 1 Bekar *t1zivil*

☐ 2 Evli, beraber yaşıyor

☐ 3 Evli, ayrı yaşıyor

☐ 4 Evli değil, bir partnerle yaşıyor

☐ 5 Boşanmış

☐ 6 Ayrı

☐ 7 Dul

18. Ana diliniz hangisidir?

Lütfen yalnızca bir kutu işaretleyiniz.

☐ 1 Türkçe *t1sprache*

☐ 2 Kürtçe

☐ 3 İsviçre Almancası / Almanca

☐ 4 Başka bir dil, yazınız ________________________ *t1sprache_off*

19. Hangi ülke vatandaşısınız?

Birden fazla ülke vatandaşı iseniz lütfen hepsini işaretleyiniz

a) İsviçre	☐ 2	*t1staata*
b) Türkiye	☐ 2	*t1staatb*
c) Vatansız	☐ 2	*t1staatc*
d) Diğer, yazınız: ________________________________	☐ 2	*t1staatd*
		t1staatd_off

20. İsviçre'ye hangi yıl geldiniz?

_______ yılında *t1inch*

21. Enson hangi okuldan mezun oldunuz?

Lütfen sadece bir kutu işaretleyiniz

- ☐ 1 hiç okula gitmedim *t1bildung*
- ☐ 2 ilk okul
- ☐ 3 orta okul
- ☐ 4 lise
- ☐ 5 üniversite ve daha yüksek

22. Şu anda çalışıyor musunuz?

Lütfen sadece bir kutu işaretleyiniz

- ☐ 1 Evet, %90 ve daha fazla *t1arbeit*
- ☐ 2 Evet, %90 ve daha az
- ☐ 3 Hayır, işsizim
- ☐ 4 Hayır, evhanımı / ev erkeğiyim
- ☐ 5 Cevap vermek istemiyorum
- ☐ 6 Hayır, Şu an eğitim yapıyorum

23. Gelirinizi nasıl elde ediyorsunuz?

Lütfen uygun olan bütün şıkları işaretleyiniz

	Evet	
a) Maaş	☐ 2	*t1einkoma*
b) Kendi işim	☐ 2	*t1einkomb*
c) Ailem, akrabalarım	☐ 2	*t1einkomc*
d) Arkadaşlarım	☐ 2	*t1einkomd*
e) Sosyal Yardım	☐ 2	*t1einkome*
f) İşsizlik sigortası	☐ 2	*t1einkomf*
g) Diğer yardımlar(Emeklilik, malüllük, vs.)	☐ 2	*t1einkomg*
h) Öğrenim kredisi	☐ 2	*t1einkomh*
i) Diğer kaynaklar	☐ 2	*t1einkomi*
j) Cevap vermek istemiyorum	☐ 2	*t1einkomj*

24. Aşağıdaki soruda sizin son 12 ay içerisinde ne kadar sıklıkta kendinizi stres altında hissetiğiniz soruluyor.

(Lütfen her soru için sadece bir kutuyu işaretleyiniz)

	çok sık	sık	bazen	nadiren	hiçbir zaman	
a) Son 12 ay içerisinde, yaşamınızdaki önemli olayları etkileyemediğinizi ne kadar sıklıkta düşündünüz?	☐4	☐3	☐2	☐1	☐0	*t1sta*
b) Son 12 ay içerisinde, işlerinizi yapma ve problemlerinizi çözme konusunda kendinizi ne kadar sıklıkta emin hissettiniz?	☐4	☐3	☐2	☐1	☐0	*t1stb*
c) Son 12 ay içerisinde, işlerin sizin istediğiniz şekilde geliştiğini ne kadar sıklıkta düşündünüz?	☐4	☐3	☐2	☐1	☐0	*t1stc*
d) Son 12 ay içerisinde, işlerinizin ve problemlerinizin çok biriktiğini ve çözemeyeceğinizi kadar sıklıkta düşündünüz?	☐4	☐3	☐2	☐1	☐0	*t1std*

25. Aşağıdaki cümleler sizin kendinizi ne kadar iyi hissediğinizle ilgili

(Lütfen her cümlede sadece bir kutuyu işaretleyiniz)

	**Tamamen** Katılıyorum				**Hiç** Katılmıyorum	
	6	5	4	3	2	1	
a) Genel olarak yaşama hakim olduğumu düşünüyorum.	☐6	☐5	☐4	☐3	☐2	☐1	*t1wba*
b) Ben günlük yaşam içinde ortaya çıkan istekler karşısında kendimin bunaldığını hissediyorum.	☐6	☐5	☐4	☐3	☐2	☐1	*t1wbb*
c) Ben çevremdeki insanlara ve topluma uyum sağlayamıyorum.	☐6	☐5	☐4	☐3	☐2	☐1	*t1wbc*
d) Ben günlük hayatda ortaya çıkan görevlerle oldukça iyi başedebiliyorum.	☐6	☐5	☐4	☐3	☐2	☐1	*t1std*
e) Benim işlerim bana fazla geliyor.	☐6	☐5	☐4	☐3	☐2	☐1	*t1wbe*
f) Yaşamımı mutlu olacağım şekilde organize etmekte zorlanıyorum.	☐6	☐5	☐4	☐3	☐2	☐1	*t1wbf*
g) Ben kendi evimden ve yarattığım yaşam tarzımdan memnunum.	☐6	☐5	☐4	☐3	☐2	☐1	*t1wbg*

Sizin alkol tüketiminiz ile ilgili sorular

26. Ne kadar sıklıkta alkollü içecek kullanıyorsunuz?
Lütfen sadece bir kutuyu işaretleyiniz

- ☐ 0 Hiçbir zaman *t1alk1*
- ☐ 1 Ayda 1 kez veya daha az
- ☐ 2 Ayda 2-4 kez
- ☐ 3 Haftada 2-4 kez
- ☐ 4 Haftada 4 veya daha fazla

27. Eğer alkol içiyorsanız, bir günde ne kadar içiyorsunuz? (=standart içecek) örneğin yaklaşık 3 dl bira (% 5 Vol.), 1 dl şarap veya köpüklü şarap (% 12,5 Vol.), 2 cl şnaps (% 55 Vol.) veya 4 cl likör (%30 Vol.).
Lütfen sadece bir kutuyu işaretleyiniz

- ☐ 0 Ben alkol içmiyorum *t1alk2*
- ☐ 1 1 veya 2
- ☐ 2 3 veya 4
- ☐ 3 5 veya 6
- ☐ 4 7 – 9
- ☐ 5 10 veya daha fazla

28. Ne kadar sıklıkta 6 veya daha fazla bardak alkol (=standart içecek) içiyorsunuz?
Lütfen sadece bir kutuyu işaretleyiniz.

- ☐ 0 Hiçbir zaman *t1alk3*
- ☐ 1 Ayda 1x daha az
- ☐ 2 Ayda 1 kez
- ☐ 3 Haftada 1 kez
- ☐ 4 Günlük veya neredeyse her gün

29. Sigarayı Bırakma Kursumuzdan nasıl haberdar oldunuz?

Lütfen uygun olan bütün şıkları işaretleyiniz

	Evet	
a) Aile/Arkadaşlar/Tanıdıklar	☐ 2	*t1aufmerka*
b) Dernekler	☐ 2	*t1aufmerkb*
c) Tiryaki Kukla Gösterimi	☐ 2	*t1aufmerkc*
d) Radyo, Televizyon (Almanca ya da Fransızca)	☐ 2	*t1aufmerkd*
e) Radyo, Televizyon (Türkçe)	☐ 2	*t1aufmerke*
f) Gazete/Dergi (Almanca ya da Fransızca)	☐ 2	*t1aufmerkf*
g) Gazete/Dergi (Türkçe)	☐ 2	*t1aufmerkg*
h) İnternet (örnek: www.stop-tabac.ch, www.at-schweiz.ch, www.tiryakikukla.ch)	☐ 2	*t1aufmerkh*
i) Sağlık Çalışanları (Doktor, Diş Hekimi, Eczane, vs.)	☐ 2	*t1aufmerki*
j) Dükkan, Restoran gibi yerlerdeki Poster, El ilanı, Broşürler	☐ 2	*t1aufmerkj*
k) Sigarayı Bırakma Yarışması	☐ 2	*t1aufmerkk*
l) Sigarayı Bırakma Hattı (Telefon Danışmanlığı)	☐ 2	*t1aufmerkl*
m) Sosyal medya (Facebook, Twitter, Instagram, WhatsApp etc.)	☐ 2	*t1aufmerkm*
n) Diğer, yazınız: ________________________________		*t1aufmerkm_off*

30. Siz, kaç kişinin bu sigara bırakma kursuna katılmasını sağladınız?

|______| kişi *t1motiviert*

31. Belirtmek istediğiniz düşünce ya da beklentileriniz var mı? *t1offen*

✎..

....

....

....

....

....

Anketimizi yanıtladığınız için teşekkür ederiz!
