# Supplementary material for: An adapted smoking-cessation intervention for Turkish-speaking migrants in Switzerland: Predictors of smoking outcomes at one-year follow-up
Source: PLoS One. 2021 Mar 18;16(3):e0247157. doi: 10.1371/journal.pone.0247157 (PMC7971503; doi:10.1371/journal.pone.0247157)
Supplement: S2 File — T1 questionnaire. (PDF) [file pone.0247157.s002.pdf]

Schweizer Institut für Sucht- und Gesundheitsforschung
Swiss Research Institute for Public Health and Addiction
Institut suisse de recherche sur la santé publique et les addictions

Evaluation of smoking cessation courses for Turkish-speaking migrants in Switzerland

T1 Questionnaire

DO NOT FILL IN! (to be filled in by the project team)

Subject number: |__ __ __ __ __ __ __ __ ____ *pbnr*

Date of the first course attendance: __ __ : __ __ : __ __ __ *t1work_day1work_mont1work_year*
DD MM YYYY

Questions on smoking behaviour

1. How often do you smoke?
Please tick only one box.

- ☐ 1 Daily *t1-frequent*
- ☐ 2 Several times per week (___ times per week) *t1-frequently2*
- ☐ 3 Once a week
- ☐ 4 Less frequently (___ times per month) *t1-frequently4*

2. How old were you when you a) ...first smoked a cigarette?
_____ years *t1ziga*
b) ... started smoking regularly?
_____ years *t1zigb*

3. How much do you generally smoke on a day that you smoke?

(a) _____ cigarettes *t1freqzigi*
(b) _____ cigars *t1freqziga*
(c) ______ Pipe *t1freq whistle*
(d) ______ hookahs *t1freqw*
(e)_______ 'joints' (hashish / 'grass' / cannabis) *t1freqjoi*
(f)_______ E-cigarettes *t1frequent*

4. How soon after waking do you smoke your first cigarette?
Please tick only one box.

- ☐ 4 Within 5 minutes *t1fag1*
- ☐ 3 Within 6 to 30 minutes
- ☐ 2 Within 31 to 60 minutes
- ☐ 1 After more than 60 minutes

5. Where and how often do you smoke at home?

Check one box in each line.

	frequently	rarely	never	
a) living room	☐ 2	☐ 1	☐ 0	*t1homea*
b) bedroom	☐ 2	☐ 1	☐ 0	*t1homeb*
c) children's room	☐ 2	☐ 1	☐ 0	*t1homec*
d) bathroom/ toilet	☐ 2	☐ 1	☐ 0	*t1homed*
e) kitchen	☐ 2	☐ 1	☐ 0	*t1homee*
f) balcony/ garden/ roof terrace	☐ 2	☐ 1	☐ 0	*t1homef*
g) Other room:______________________________	☐ 2	☐ 1	☐ 0	*t1homegt1homeg_off*

6. Smoking in a car: Do you or someone living with you own a car?

☐ 1 No , *t1autoa*

☐ 2 Yes

	frequently	rarely	never	
a) If so, how often do you or other passengers/drivers smoke in the car?	☐ 2	☐ 1	☐ 0	*t1autob*

7. How many people live with you in the same household? (including yourself)

Please enter the number: ⎿____⏌ Persons living in my household *t1mitbewo*

8. How many people who live with you smoke? (including yourself)

Please enter the number: ⎿____⏌ people smoke *t1withbewora*

9. Do you have children who live in the same household as you?

☐ 2 Yes *t1kind*

☐ 1 No

(a) If so, how many children live with you ⎿____⏌ *t1anzki*

(b) Age of the children ⎿____⏌ ⎿____⏌ ⎿____⏌ ⎿____⏌ ⎿____⏌ *t1altki1-5*

1st child child2 child3 child4 child5

10. How many of your 10 most important family members and friends in Switzerland smoke cigarettes?

Please enter the number: └──┘ people smoke *t1people*

11. What is your general opinion of smoking? What do you think is best?

Check one box in each line. There are no right and wrong answers. Just express your opinion.

		I completely agree	I somewhat agree	I somewhat disagree	I disagree completely	
		1	2	3	4	
a)	Smoking helps against boredom.	☐1	☐2	☐3	☐4	*t1ea*
b)	Smoking leaves an unpleasant smell.	☐1	☐2	☐3	☐4	*t1eb*
c)	Smoking underlines a modern attitude.	☐1	☐2	☐3	☐4	*t1ec*
d)	Smoking makes the skin age faster.	☐1	☐2	☐3	☐4	*t1ed*
e)	Smoking calms and relaxes.	☐1	☐2	☐3	☐4	*t1e*
f)	Smoking is harmful to other people's health.	☐1	☐2	☐3	☐4	*t1ef*
g)	Smoking tastes good.	☐1	☐2	☐3	☐4	*t1eg*
h)	What is your opinion on the general ban on smoking in public places, restaurants, cafés and bars?	☐1	☐2	☐3	☐4	*t1eh*

Questions on smoking cessation

12. Have you ever tried to quit smoking before the stop smoking course?

☐1 No *t1stop*

☐2 Yes

If so, how often? _____ Times *t1anzstop*

13. How strong is your readiness to quit smoking at this time?

Please circle the number that corresponds to your estimation:

t1stoptermo

0 —— 1—— 2 —— 3 —— 4 —— 5 —— 6 —— 7 —— 8 —— 9 —— 10

0 = not ready at all 10 = I am very ready

14. What are your expectations to be smoke-free after one year, with the help of this stop smoking course?

Please circle the number that corresponds to your estimation:

t1expectation

0 —— 1—— 2 —— 3 —— 4 —— 5 —— 6 —— 7 —— 8 —— 9 —— 10

0 = not confident at all 10 = I am very confident

Questions about you

15. Are you a man or a woman?

☐ 1 man t1sex
☐ 2 woman

16. How old are you? *t1 age*

____________ years old

17. Are you currently...?
Please tick only one box.

☐ 1 Single *t1civil*
☐ 2 Married, living together
☐ 3 Married, living separately
☐ 4 Not married, living in a stable partnership
☐ 5 Divorced
☐ 6 Separate
☐ 7 Widowed

18. What is your mother tongue?
Please tick only one box.

☐ 1 Turkish *t1 language*
☐ 2 Kurdish
☐ 3 Swiss German/German
☐ 4 Other language: _________________________ *t1 language_off*

19. What is your citizenship?
Please tick all that apply

(a) Switzerland ☐ 2 *t1staata*
(b) Turkey ☐ 2 *t1stateb*
(c) None ☐ 2 *t1staatc*
(d) Other: ___________________________________ ☐ 2 *t1statdt1statd_off*

20. When did you come to Switzerland (year)?

In year ________ *t1inch*

21. What is the highest degree or level of education you have completed?

Please tick only one box.

- ☐ 1 No school *t1 formation*
- ☐ 2 Primary school (7-12 years)
- ☐ 3 Middle school (10-12 years)
- ☐ 4 Secondary school (15-18 years)
- ☐ 5 College (university)

22. Are you working at the moment?

Please tick only one box.

- ☐ 1 Yes, I work full time (90% and more) *t1work*
- ☐ 2 Yes, I work part time (less than 90%)
- ☐ 3 No, I am unemployed.
- ☐ 4 No, I am exclusively a housewife/househusband.
- ☐ 5 I don't want to say anything about that.
- ☐ 6 No, I am currently in training/school.

23. Where do you currently draw your income from?

Please tick all that apply

	Applicable	
(a) paid labour	☐ 2	*t1incoma*
(b) self-employment	☐ 2	*t1incomb*
(c) family, kinship	☐ 2	*t1einkomc*
(d) friends	☐ 2	*t1incoming*
(e) welfare, social welfare office, relief agency	☐ 2	*t1income*
(f) unemployment benefit	☐ 2	*t1income*
(g) other benefits (pensions, IV, etc.)	☐ 2	*t1income*
(h) training allowances	☐ 2	*t1incoming*
(i) Other sources	☐ 2	*t1einkomi*
(j) I don't want to say anything about that.	☐ 2	*t1einkomj*

24. The following questions deal with how often you have been under stress in the last 12 months. (Please tick one answer per statement)

	Very often	frequently	sometimes	rarely	never	
a) In the last 12 months, how often have you had the feeling that you were unable to influence important things in your life?	☐4	☐3	☐2	☐1	☐0	*t1sta*
b) How often in the last 12 months have you felt confident in dealing with your personal tasks and problems?	☐4	☐3	☐2	☐1	☐0	*t1stb*
c) In the last 12 months, how often have you had the feeling that things are going according to your expectations?	☐4	☐3	☐2	☐1	☐0	*t1stc*
d) In the last 12 months, how often have you had the feeling that tasks or problems have accumulated to such an extent that you cannot cope with them?	☐4	☐3	☐2	☐1	☐0	*t1std*

25. The following statements deal with how well you feel (Please tick one answer per statement)

	Totally true				Not applicable at all		
	6	5	4	3	2	1	
a) In general, I feel that I have life under control.	☐6	☐5	☐4	☐3	☐2	☐1	*t1wba*
b) I am often overwhelmed with the demands of everyday life.	☐6	☐5	☐4	☐3	☐2	☐1	*t1wbb*
c) I do not fit in well with the people and society around me.	☐6	☐5	☐4	☐3	☐2	☐1	*t1wbc*
d) I can handle everyday tasks quite well.	☐6	☐5	☐4	☐3	☐2	☐1	*t1std*
e) My tasks often overwhelm me.	☐6	☐5	☐4	☐3	☐2	☐1	*t1wbe*
f) I find it difficult to organize my life in a way that I am satisfied with it.	☐6	☐5	☐4	☐3	☐2	☐1	*t1wbf*
g) I am very satisfied with the housing situation and the lifestyle I have created for myself.	☐6	☐5	☐4	☐3	☐2	☐1	*t1wbg*

Questions about your alcohol consumption

26. How often do you consume alcoholic beverages?
Please tick only one box.

☐ 0 Never *t1alk1*
☐ 1 1 x per month or less
☐ 2 2 - 4 times a month
☐ 3 2 - 4 x per week
☐ 4 4 x or more per week

27. If you drink alcoholic beverages, how much do you typically drink in a day? An alcoholic drink (= standard drink) corresponds, for example, to approx. 3 dl beer (5 vol.%), 1dl wine or sparkling wine (12.5 vol.%), 2 cl schnapps (55 vol.%) or 4 cl liqueur (30 vol.%).
Please tick only one box.

☐ 0 I don't drink alcohol. *t1alk2*
☐ 1 1 or 2
☐ 2 3 or 4
☐ 3 5 or 6
☐ 4 7 - 9
☐ 5 10 or more

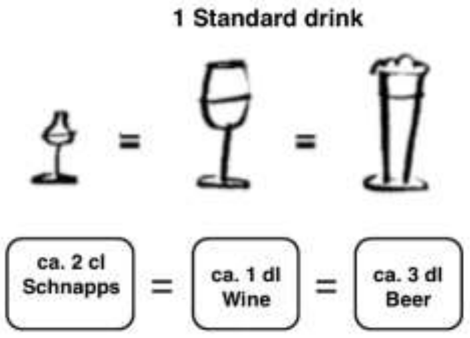

28. How often do you drink 6 or more glasses of alcohol (= standard drink) on one occasion?
Please tick only one box.

☐ 0 Never *t1alk3*
☐ 1 Less than 1x per month
☐ 2 1 x per month
☐ 3 1 x per week
☐ 4 Daily or almost daily

Questions about the course

29. How did you become aware of our smoking cessation course?

Please tick all that apply

	Applicable	
(a) Family/ friends/ acquaintances	☐ 2	*t1aufmerka*
(b) Clubs	☐ 2	*t1watchful*
(c) Tiryaki Kukla Event	☐ 2	*t1attentionc*
(d) Radio, television (in German or French)	☐ 2	*t1remarking*
(e) Radio, television (in Turkish)	☐ 2	*t1observations*
(f) Newspapers/ magazines (in German or French)	☐ 2	*t1attention*
(g) Newspapers/ magazines (in Turkish language)	☐ 2	*t1attention*
(h) Internet (e.g. www.stop-tabac.ch, www.at-schweiz.ch, consulate)	☐ 2	*t1attention*
(i) health professionals (doctor, dentist, pharmacist, specialised institution, etc.)	☐ 2	*t1aufmerki*
(j) Posters, flyers, brochures in shops, restaurants etc.	☐ 2	*t1attentionj*
(k) anti-smoking competition	☐ 2	*t1attention*
(l) Smoke Stop Line (smokers' telephone)	☐ 2	*t1attention*
(m) Social media (Facebook, Twitter, Instagram, WhatsApp etc.	☐ 2	*t1attribution km*
(n) Other: ________________________________		*t1uperkm_off*

30. How many people were you able to persuade to take part in this stop smoking course?

└──────┘ number of people *t1motivated*

31. Do you have wishes and expectations that you would share with us? ***t1open***

✐..

....

....

Many thanks for your cooperation!
