# Supplementary material for: An adapted smoking-cessation intervention for Turkish-speaking migrants in Switzerland: Predictors of smoking outcomes at one-year follow-up
Source: PLoS One. 2021 Mar 18;16(3):e0247157. doi: 10.1371/journal.pone.0247157 (PMC7971503; doi:10.1371/journal.pone.0247157)
Supplement: S3 File — Sigara İçmeyenler İçin T2 anketi. (PDF) [file pone.0247157.s003.pdf]

Schweizer Institut für Sucht- und Gesundheitsforschung
Swiss Research Institute for Public Health and Addiction
Institut suisse de recherche sur la santé publique et les addictions

Kamu Sağlığı ve Bağımlılık Araştırma Enstitüsü

İsviçre'de Yaşayan Türkçe Konuşan Göçmenler İçin Sigarayı Bırakma Kursu Değerlendirme Anketi

Sigara İçmeyenler İçin T2 anketi

LÜTFEN DOLDURMAYIN! (Proje yetkilileri dolduracaktır)

Kurs-Nummer |________| *kurscode*

Probanden-Nummer: |__ __ __ __ __ __ __ ____| *pbnr*

Datum des letzten Kurbesuchs:

__ __ : __ __ : __ __ __ __ *t2ku_tag* *t2ku_mon* *t2ku_jah*
T T M M J J J J

Datum, an dem FB T2 ausgefüllt wurde

__ __ : __ __ : __ __ __ __ *t2fb_tag* *t2fb_mon* *t2fb_jah*
T T M M J J J J

Katılımla İlgili Sorular

1. Hiç bir seansı kaçırmadan bütün kursa katıldınız mı?

☐ 2 Evet, hiç bir seansı kaçırmadım ve hepsine katıldım. *ohneabs*

☐ 1 Hayır, en az bir seansı kaçırdım.
Kaç seansa katılamadınız? _____ Seans *freqabs*

Sigara Bırakma İle İlgili Sorular

2. Ne zamandan beri hiç sigara içmiyorsunuz?

__ __ : __ __ : __ __ __ __ **(tam olarak bilemiyorsanız,** *betag, bemon, bejahr*
G G A A Y Y Y Y **yaklaşık bir tarih veriniz)**

3. Sigarayı bırakma girişiminizde nikotin içren ürünlerden ya da başka metotlardan faydalandınız mı?

Lütfen her satırda bir kutu işaretleyiniz.

	Evet	Hayır	
a) Nikotin-Bantı	☐ 2	☐ 1	*t2hilfa*
b) Nikotin-Sakızı	☐ 2	☐ 1	*t2hilfb*
c) Dil altı Nikotin Tabletleri	☐ 2	☐ 1	*t2hilfc*
d) „Zyban“	☐ 2	☐ 1	*t2hilfd*
e) „Champix“	☐ 2	☐ 1	*t2hilfe*
f) Akupunktur	☐ 2	☐ 1	*t2hilff*
g) Hipnoz	☐ 2	☐ 1	*t2hilfg*
h) Diğer, yazınız: ______________________________	☐ 2	☐ 1	*t2hilfh* *t2hilfh_off*
i) Nicorette® ağızlık	☐ 2	☐ 1	*t2hilfi*
j) Nicorette® Sprey	☐ 2	☐ 1	*t2hilfj*
k) Sigarayı Bırakma Telefon Hattı	☐ 2	☐ 1	*t2hilfk*

4. Bir daha sigara içmemek için kendinizi ne kadar hazır hissediyorsunuz?
Lütfen kararınıza uygun sayıyı yuvarlak içine alınız:

0 — 1— 2 — 3 — 4 — 5 — 6 — 7 — 8 — 9 — 10 *bereitzuk*

0 = Hiç hazır değilim - Tekrar içmek istiyorum

10 = Çok hazırım bir daha sigara içmeyeceğim

5. Genel olarak sigara konusunda ne düşünüyorsunuz? Görüşlerinize en iyi hangisi uyuyor? *Lütfen her satırda bir kutu işaretleyiniz. Doğru ya da yanlış cevap yoktur. Sadece düşüncenizi belirtiniz.*

		**Tamamen katılıyorum**	**Biraz katılıyorum**	**Pek katılmıyorum**	**Hiç katılmıyorum**	
		1	2	3	4	
a)	Sigara can sıkıntısına iyi gelir	☐	☐	☐	☐	*t2ea*
b)	Sigara ardında kötü bir koku bırakır.	☐	☐	☐	☐	*t2eb*
c)	Sigara içmek modern bir görünüş sağlar.	☐	☐	☐	☐	*t2ec*
d)	Sigara cildi daha çabuk yaşlandırır.	☐	☐	☐	☐	*t2ed*
e)	Sigara içmek sakinleştirir.	☐	☐	☐	☐	*t2ee*
f)	Sigara başkalarının sağlığını bozar.	☐	☐	☐	☐	*t2ef*
g)	Sigaranın tadı güzeldir.	☐	☐	☐	☐	*t2eg*
h)	Restorant, kahve ve bar gibi kamuya açık yerlerde sigara içilmesinin yasaklanması konusunda ne düşünüyorsunuz?	☐	☐	☐	☐	*t2eh*

6. Sigara içme isteğiniz var, fakat sigara içmek istemiyorsunuz: Sigara içmemek için, ne yapabilirsiniz? (als OFFENE Frage stellen, TN müssen frei antworten > Ablenkung vom Craving mit Gedanken, Mund und Händen)

☐ *t2alt1* Kitap/ gazete okurum

☐ *t2alt2* Yürüyüş, spor yaparım, hareket ederim

☐ *t2alt3* Sakız çiğnerim

☐ *t2alt4* Televizyo, bilgisayar

☐ *t2alt5* Meyve/ sebze yerim

☐ *t2alt6* Su içerim

☐ *t2alt7* Sigara içilen yerlerden uzak dururum

☐ *t2alt8* Ev işleri yaparım (çamaşır/ temizlik)

☐ *t2alt9* Uyurum, dinlenirim

☐ *t2alt10* Başka şeyler: ___________________

7. Sigaranın içinde blunan, sağlığa zarar veren 3 önemli maddeyi ve sağlığa nasıl zarar verdiğini belirtiniz.
(als OFFENE Frage stellen, TN müssen frei antworten)

	Sigaranın içindeki 3 zararlı madde		Bu maddelerin sağlığa zararları
☐ *t2inh1*	Nikotin	☐ *t2inh1a*	Bağımlılık yapar
☐ *t2inh2*	Katran	☐ *t2inh2a*	Kanser yapar, solunum yollarına ve akcigere zarar verir
☐ *t2inh3*	Karbonmonoksit	☐ *t2inh3a*	Kalp krizi, beyin kanaması, nefes darlığı
☐ *t2inh4*	Diğeri: ___________________	☐ *t2inh4a*	Diğeri: _________________________

Çevrenizde sigara içilen alanlarla ile ilgili sorular

8. Evinizde nerelerde ve ne kadar sıklıkta sigara içiliyor?
Her satırda bir kutu işaretleyiniz.

	sık sık	nadiren	hiç	
a) Oturma odası	☐ 2	☐ 1	☐ 0	*t2homea*
b) Yatak odası	☐ 2	☐ 1	☐ 0	*t2homeb*
c) Çocuk odası	☐ 2	☐ 1	☐ 0	*t2homec*
d) Banyo / Tuvalet	☐ 2	☐ 1	☐ 0	*t2homed*
e) Mutfak	☐ 2	☐ 1	☐ 0	*t2homee*
f) Balkon / Bahçe / Teras	☐ 2	☐ 1	☐ 0	*t2homef*
g) Başka yer (yazınız):__________________________	☐ 2	☐ 1	☐ 0	*t2homeg* *t2homeg_off*

9. Arabada sigara içmek: Sizin veya birlikte yaşadığınız kişinin arabası var mı?
☐ 1 Hayır *t2autoa*
☐ 2 Evet

	sık sık	nadiren	hiç	
a) Eğer cevabınız evetse, ne kadar sıklıkla bu arabada sigara içiliyor?	☐ 2	☐ 1	☐ 0	*t2autob*

10. Evinizde kaç kişi yaşıyor? (siz dahil)

Lütfen sayıyı yazınız: |______| kişi yaşıyor *t2mitbewo*

11. Birlikte yaşadığınız kişilerden kaçı sigara içiyor?

Lütfen sayıyı yazınız: |______| kişi *t2mitbewora*

12. İsviçre'deki aile ve arkadaşlarınızdan en yakınınızdaki 10 kişiden kaçı sigara içiyor?

Lütfen sayısını yazınız: |______| kişi *t2personen*

13. Çeverenizde, sizin sigara braktığınızdan etkilenerek, sigara bırakan kişiler oldu mu

☐ 1 Hayır *t2andere*
☐ 2 Evet

Eğer evetse, bu kişil kimler?

		**Bu kişiler hala sigara içmiyor mu?**		
1 ________________	*t2p1*	☐ 1 Hayır	☐ 2 Evet	*t2rf1*
2 ________________	*t2p2*	☐ 1 Hayır	☐ 2 Evet	*t2rf2*
3 ________________	*t2p3*	☐ 1 Hayır	☐ 2 Evet	*t2rf3*
4 ________________	*t2p4*	☐ 1 Hayır	☐ 2 Evet	*t2rf4*

Kursla ilgili sorular

14. Sizinde katılığınız sigara kursunu organize eden dernek veya cami ile hala ilişkiniz var mı?

☐ 1 Hayır ☐ 2 Evet *t2verein*

Eğer cevabınız evetse, bu dernek ve camide sigara / sigara bırakma konuları güncelmi ve herhangi bir etkinlik düzenlendi mi?

☐ 1 Hayır konu güncel değil ve bu konuda herhangi bir etkinlik düzenlemedi *t2impact*

☐ 2 Bilmiyorum

☐ 3 Evet, sigara konusu üzerine öncekinden daha fazla konuşuluyor

☐ 4 Evet, hala sigarayı bırakma ile ilgilenen başka kişiler var

☐ 5 Evet, bu konuyla ilgili etkinlikler düzenleniyor-hangi etkinlik? *t2aktion*

____

15. Kurs, sigarayı bırakma denemenizde size yardımcı oldu mu?
Lütfen sadece bir kutu işaretleyiniz.

- ☐ 1 Çok yardımcı oldu *hilfver*
- ☐ 2 Biraz yardımcı oldu
- ☐ 3 Şöyle böyle
- ☐ 4 Pek yardımcı olmadı
- ☐ 5 Hiç yardımcı olmadı

16. Sigarayı bırakma kursu size ve diğerlerine başka alalarda da (günlük konular, ilişki, grup içinde öğrenmek vb.) yardımcı oldu mu?
Lütfen sadece bir kutu işaretleyiniz.

- ☐ 1 Çok yardımcı oldu *hilfges*
- ☐ 2 Biraz yardımcı oldu
- ☐ 3 Şöyle böyle
- ☐ 4 Pek yardımcı olmadı
- ☐ 5 Hiç yardımcı olmadı

17. Sigara Bırakma Kursunu çevrenizde ilgi duyan insanlara tavsiye eder misiniz?
Lütfen sadece bir kutu işaretleyiniz.

- ☐ 1 Evet, kesinlikle ederim *t2empfehl*
- ☐ 2 Herhalde ederim
- ☐ 3 Bilmiyorum
- ☐ 4 Herhalde etmem
- ☐ 5 Hayır, kesinlikle etmem

18. Kursun daha iyi olması için önerilerinizvar mı?

✎ *t2offen*

....

....

....

Anketi doldurduğunuz için teşekkür ederiz!
