# Supplementary material for: An adapted smoking-cessation intervention for Turkish-speaking migrants in Switzerland: Predictors of smoking outcomes at one-year follow-up
Source: PLoS One. 2021 Mar 18;16(3):e0247157. doi: 10.1371/journal.pone.0247157 (PMC7971503; doi:10.1371/journal.pone.0247157)
Supplement: S4 File — T2 questionnaire for non-smokers. (PDF) [file pone.0247157.s004.pdf]

Evaluation of stop smoking courses for Turkish-speaking migrants in Switzerland

T2 questionnaire for non-smokers

DO NOT FILL IN! (will be filled in by the project team)

Course number|________| *course code*

Subject-number: |__ __ __ __ __ __ __ ____| *pbnr*

Date of the last course attendance:

__ __ : __ __ : __ __ __ __ *t2ku_tagt2ku_mont2ku_jah*

Day Month Year

Date on which FB T2 was filled in

__ __ : __ __ : __ __ __ __ *t2fb_tagt2fb_mont2fb_jah*

Day Month Year

Questions about participation

1. Did you take part in the entire stop smoking course without skipping a session?

☐2 Yes, I never missed a session and participated in all of them *withoutabs*

☐1 No, I missed at least one session.

How many sessions did you miss? ____ sessions *freqabs*

Questions about the smoke stop

2. When did you stop smoking cigarettes?

since: __ __ : __ __ : __ __ __ __ **(if not known exactly anymore, enter approximate date)** *day, bemon, year*

Day Month Year

3. Have you used nicotine-containing drugs/preparations in connection with the smoking cessation or have you been treated with other methods?

Check one box in each line

	Applicable	Not applicable	
(a) Nicotine skin patches	☐2	☐1	*t2hilfa*
(b) Nicotine gum	☐2	☐1	*t2helpb*
(c) Nicotine-containing tablets for under the tongue	☐2	☐1	*t2helpc*
(d) Drug product "Zyban®".	☐2	☐1	*t2helpd*
(e) Drug product "Champix®".	☐2	☐1	*t2help*
(f) Acupuncture	☐2	☐1	*t2hilff*
(g) Hypnosis	☐2	☐1	*t2hilfg*
(h) Other: ________________________________	☐2	☐1	*t2helph* *t2helph_off*
(i) Nicorette® Inhaler	☐2	☐1	*t2hilfi*
(j) Nicorette® Spray	☐2	☐1	*t2helpj*
(k) smoke stop line	☐2	☐1	*t2help*

4. How strong is your readiness to stop smoking in the future?

Please circle the number that corresponds to your estimation:

0 — 1— 2 — 3 — 4 — 5 — 6 — 7 — 8 — 9 — 10

ready for

0 = no readiness - I want to continue smoking

10 = very much ready not to smoke again

5. What is your general opinion about smoking? What do you think is best?

Check one box in each row. There are no right and wrong answers. Just express your opinion.

		**I completely agree**	**I somewhat agree**	**I somewhat disagree**	**I disagree completely**	
a)	Smoking helps against boredom.	☐1	☐2	☐3	☐4	*t2ea*
b)	Smoking leaves an unpleasant smell.	☐1	☐2	☐3	☐4	*t2eb*
c)	Smoking underlines a modern attitude.	☐1	☐2	☐3	☐4	*t2ec*
d)	Smoking makes the skin age faster.	☐1	☐2	☐3	☐4	*t2ed*
e)	Smoking calms and relaxes.	☐1	☐2	☐3	☐4	*t2ee*
f)	Smoking damages the health of other people.	☐1	☐2	☐3	☐4	*t2ef*
g)	Smoking tastes good.	☐1	☐2	☐3	☐4	*t2eg*
h)	What is your opinion on the general ban on smoking in public places, restaurants, cafés and bars?	☐1	☐2	☐3	☐4	*t2eh*

6. If you feel like smoking a cigarette, but do not want to smoke: What can you do instead, not to smoke? *(ask as an OPEN question, participant must answer freely > distraction from craving with thoughts, mouth and hands)*

- ☐ *t2alt1* Read a book/newspaper
- ☐ *t2alt2* Walking, sports, exercise
- ☐ *t2alt3* Chewing gum
- ☐ *t2alt4* TV, Internet
- ☐ *t2alt5* Eating fruit/ vegetables
- ☐ *t2alt6* Drink water
- ☐ *t2alt7* Avoid places where people smoke
- ☐ *t2alt8* Household activities (washing/cleaning)
- ☐ *t2alt9* Sleep, relax
- ☐ *t2alt10* Other: ________________________

7. **Please name the 3 most important ingredients of a cigarette and their effects on your health** *(as an OPEN question, TN must answer freely)*

	Ingredient		Impact on health
☐ *t2inh1*	Nicotine	☐ *t2inh1a*	Addiction
☐ *t2inh2*	Tar	☐ *t2inh2a*	Cancer, affects respiratory tract & lungs
☐ *t2inh3*	Carbon Monoxide	☐ *t2inh3a*	Heart attack, stroke, decreased oxygen transport
☐ *t2inh4*	Other: ____________________	☐ *t2inh4a*	Other: ___________________________

Questions about smoking in your environment

8. **Where and how often do you smoke at home?**
Check one box in each row.

	frequently	rarely	never	
a) living room	☐2	☐1	☐0	*t2homea*
b) bedroom	☐2	☐1	☐0	*t2homeb*
c) children's room	☐2	☐1	☐0	*t2homec*
d) bath / Toilet	☐2	☐1	☐0	*t2homed*
e) kitchen	☐2	☐1	☐0	*t2homee*
f) balcony / garden / roof terrace	☐2	☐1	☐0	*t2homef*
g) other room: ___________________________________	☐2	☐1	☐0	*t2homeg* *t2homeg_off*

9. **Smoking in a car: Do you or someone who lives with you own a car?**

☐ 1 No *t2autoa*
☐ 2 Yes

	frequently	rarely	never	
a) If so, how often do you or other passengers/drivers smoke in the car?	☐2	☐1	☐0	*t2car*

10. **How many people live with you in the same household? (including yourself)**

Please enter the number: |______| Persons living in my household *t2mitbewo*

11. How many people who live with you smoke?

Please enter the number: ⌊______⌋ people smoke *t2mitbewora*

12. How many of your 10 most important family members and friends in Switzerland smoke cigarettes?

Please enter the number: ⌊______⌋ people smoke *t2people*

13. Has anyone close to you also tried to quit smoking because you have stopped?

☐ 1 No *t2other*
☐ 2 Yes

		Are these people today still smoke-free?	
If so, who?	1__________________ *t2p1*	☐ 1 No ☐Yes	*t2rf1*
	2__________________ *t2p2*	☐ 1 No ☐Yes	*t2rf2*
	3__________________ *t2p3*	☐ 1 No ☐Yes	*t2rf3*
	4__________________ *t2p4*	☐ 1 No ☐Yes	*t2rf4*

Questions about the stop smoking course

14. Do you still have contact with the club/mosque that organized the stop smoking course at the time? *(do not ask in informal groups)*

☐ 1 No ☐Yes *t2club*

If YES, is the topic smoking/smoking cessation in your club or mosque still up to date and were any activities carried out in addition?

☐1 No, the topic is no longer current and there were no activities on it. *t2impact*
☐2 I don't know.
☐3 Yes, there is still more talk about smoking than before.
☐4 Yes, there are other people interested in being smoke-free.
☐5 Yes, there were activities related to it - Which ones? *t2 action*
____

15. Was the stop smoking course helpful for you in trying to quit smoking?

Please tick only one box.

- ☐1 Very helpful *help*
- ☐2 Rather helpful
- ☐3 Neither nor
- ☐4 Rather not helpful
- ☐5 Not helpful at all

16. Was the stop smoking course helpful for you in other areas as well (support with everyday questions, contact, learning in the group etc.)?

Please tick only one box.

- ☐1 Very helpful *auxiliary*
- ☐2 Rather helpful
- ☐3 Neither nor
- ☐4 Rather not helpful
- ☐5 Not helpful at all

17. Would you recommend this smoking cessation course to interested people from your environment?

Please tick only one box.

- ☐1 Yes, for sure *t2recommendation*
- ☐2 Probably yes
- ☐3 I don't know
- ☐4 Probably no
- ☐5 No, certainly not

18. Do you have any further suggestions for the improvement of the course?

✎ *t2open*

....

....

....

Many thanks for your cooperation !
