# Supplementary material for: An adapted smoking-cessation intervention for Turkish-speaking migrants in Switzerland: Predictors of smoking outcomes at one-year follow-up
Source: PLoS One. 2021 Mar 18;16(3):e0247157. doi: 10.1371/journal.pone.0247157 (PMC7971503; doi:10.1371/journal.pone.0247157)
Supplement: S5 File — Sigara içenler için T2 Anketi. (PDF) [file pone.0247157.s005.pdf]

Schweizer Institut für Sucht- und Gesundheitsforschung
Swiss Research Institute for Public Health and Addiction
Institut suisse de recherche sur la santé publique et les addictions

Kamu Sağlığı ve Bağımlılık Araştırma Enstitüsü

İsviçre'de Yaşayan Türkçe Konuşan Göçmenler İçin Sigarayı Bırakma Kursu Değerlendirme Anketi

Sigara içenler için T2 Anketi

LÜTFEN DOLDURMAYIN! (YETKİLİLER TARAFINDAN DOLDURULACAKTIR)

Kurs-Nummer |________| *kurscode*

Probanden-Nummer: |__ __ __ __ __ __ __ ____| *pbnr*

Datum des letzten Kurbesuchs:

__ __ : __ __ : __ __ __ __ *t2ku_tag* *t2ku_mon* *t2ku_jah*
T T M M J J J J

Datum, an dem FB T2 ausgefüllt wurde

__ __ : __ __ : __ __ __ __ *t2fb_tag* *t2fb_mon* *t2fb_jah*
T T M M J J J J

Başlangıç soruları

1. Hiç bir seansı kaçırmadan bütün kursa katıldınız mı?

☐ 2 Evet, hiç bir seansı kaçırmadım ve hepsine katıldım. *ohneabs*

☐ 1 Hayır, en az bir seansı kaçırdım.
Kaç seansa katılamadınız? _____ Seans *freqabs*

2. Geçen 4 ay içinde hiç sigarayı bıraktınız mı?

☐ 1 Hayır *t2stop*
☐ 2 Evet, bir kez
☐ 3 Evet, birden fazla

➡ **Geçen 4 ay içerisinde sigarayı bıraktıysanız (ancak yeniden başladıysanız) → lütfen <u>3. Sorudan</u> devam ediniz**

➡ **Geçen 4 ay içinde sigarayı <u>hiç bırakmadıysanız</u> →lütfen <u>5. Sorudan</u> devam ediniz**

Sigara bırakma ile ilgili sorular – yalnızca en az bir kere sigarayı bırakanlar (ancak tekrar başlayanlar) için

3. Son 4 ay içinde en uzun kaç gün arka arkaya sigara içmediniz (Hastalık ya da hastanede yatmaktan dolayı hariç)?

|__ __ __| **Gün** **(tam olarak hatırlayamıyorsanız, yaklaşık olarak yazınız)** *dauerstop*

__ __ : __ __ : __ __ __ __ tarihinden **(tam olarak hatırlayamıyorsanız, yaklaşık bir tarih yazınız)** *betag, bemon, bejahr*
G G A A Y Y Y Y

__ __ : __ __ : __ __ __ __ tarihine kadar **(tam olarak hatırlayamıyorsanız, yaklaşık bir tarih yazınız)** *endtag, endmon, endjahr*
G G A A Y Y Y Y

4. Sigarayı bırakma girişiminizde nikotin içren ürünlerden ya da başka metotlardan faydalandınız mı?

Lütfen her satırda bir kutu işaretleyiniz

	Evet	Hayır	
a) Nikotin-Bantı	☐ 2	☐ 1	*t2hilfa*
b) Nikotin-Sakızı	☐ 2	☐ 1	*t2hilfb*
c) Dil altı Nikotin Tabletleri	☐ 2	☐ 1	*t2hilfc*
d) „Zyban“	☐ 2	☐ 1	*t2hilfd*
e) „Champix“	☐ 2	☐ 1	*t2hilfe*
f) Akupunktur	☐ 2	☐ 1	*t2hilff*
g) Hipnoz	☐ 2	☐ 1	*t2hilfg*
h) Diğer, yazınız: ________________________________	☐ 2	☐ 1	*t2hilfh* *t2hilfh_off*
i) Nicorette® Ağızlık	☐ 2	☐ 1	*t2hilfi*
j) Nicorette® Sprey	☐ 2	☐ 1	*t2hilfj*
k) Sigarayı Bırakma Telefon Hattı	☐ 2	☐ 1	*t2hilfk*

Sigara içme davranışları ile ilgili sorular (tüm katılımcılar)

5. Ne kadar sıklıkta sigara içiyorsunuz?
Lütfen sadece bir kutu işaretleyiniz

- ☐ 1 Hergün *t1häufig*
- ☐ 2 Haftada birden fazla (haftada ___ kez) *t1häufig2*
- ☐ 3 Haftada bir kez *t1häufig3*
- ☐ 4 Daha az (ayda ___ kez) *t1häufig4*

6. Sigara içtiğiniz bir günde, genel olarak kaç tane sigara içiyorsunuz?

a) Günde _______ tane sigara *t1freqzigi*
b) Günde _______ tane puro *t1freqziga*
c) Günde _______ kere pipo *t1freqpfei*
d) Günde ________ kere nargile *t1freqw*
e) Günde ________ tane "Joint" (Haşhaş / „Ot" / Esrar) *t1freqjo*
f) Günde ________ tane e-sigara *t1freqezig*

7. Normalde sabah kalktıktan ne kadar sonra ilk sigaranızı içiyorsunuz?
Sadece bir kutu işaretleyiniz.

- ☐ 4 5 dakika içinde *t2fag1*
- ☐ 3 6 - 30 dakika sonra
- ☐ 2 31 - 60 dakika sonra
- ☐ 1 60 dakikadan fazla

8. Evinizde nerelerde ve ne kadar sıklıkta sigara içiliyor?

Her satırda bir kutuyu işaretleyiniz.

	sık sık	nadiren	hiç	
a) Oturma odası	☐ 2	☐ 1	☐ 0	*t2homea*
b) Yatak odası	☐ 2	☐ 1	☐ 0	*t2homeb*
c) Çocuk odası	☐ 2	☐ 1	☐ 0	*t2homec*
d) Banyo / Tuvalet	☐ 2	☐ 1	☐ 0	*t2homed*
e) Mutfak	☐ 2	☐ 1	☐ 0	*t2homee*
f) Balkon / Bahçe / Teras	☐ 2	☐ 1	☐ 0	*t2homef*
g) Başka yer (yazınız):__________________________	☐ 2	☐ 1	☐ 0	*t2homeg* *t2homeg_off*

9. Arabada sigara içmek: Sizin veya birlikte yaşadığınız kişinin arabası var mı?

☐ 1 Hayır *t2autoa*

☐ 2 Evet

	sık sık	nadiren	hiç	
a) Eğer cevabınız evetse, ne kadar sıklıkta bu arabada sigara içiliyor?	☐ 2	☐ 1	☐ 0	*t2autob*

10. Evinizde kaç kişi yaşıyorsunuz? (Siz dahil)

Lütfen sayısını yazınız: └──────┘ kişi yaşıyoruz. *t2mitbewo*

11. Sizinle aynı evde yaşayanlardan kaçı sigara içiyor? (Siz dahil)

Lütfen sayıyı yazınız: └──────┘ kişi *t2mitbewora*

12. İsviçre'deki aile ve arkadaşlarınızdan en yakınınızdaki 10 kişiden kaçı sigara içiyor?

Lütfen sayıyı yazınız: └──────┘ kişi *t2personen*

13. Çeverenizde, sizin sigara braktığınızdan etkilenerek, sigara bırakan kişiler oldu mu?

t2ande

☐ 1 Hayır
☐ 2 Evet

			Bu kişiler hala sigara içmiyor mu?		
**Eğer evetse, bu kişiler kimler?**	1 ________________	t2p1	☐ 1 Hayır	☐ 2 Evet	t2rf1
	2 ________________	t2p2	☐ 1 Hayır	☐ 2 Evet	t2rf2
	3 ________________	t2p3	☐ 1 Hayır	☐ 2 Evet	t2rf3
	4 ________________	t2p4	☐ 1 Hayır	☐ 2 Evet	t2rf4

14. Neden hala sigara içiyorsunuz?

Lütfen her satırda bir kutu işaretleyiniz.

		Doğru	Yanlış	
		2	1	
a)	Arkadaşlarım sigara içiyor	☐	☐	grunda
b)	Eşim / Partnerim sigara içiyor	☐	☐	grundb
c)	Bırakmayı devamlı erteliyorum	☐	☐	grundc
d)	Bırakmak çok zor / sigarayı nasıl bırakacağımı hala bilemiyorum	☐	☐	grundd
e)	Bırakmak istemiyorum	☐	☐	grunde
f)	Başka nedenler, yazınız: ________________________	☐	☐	grundf grundf_off

15. Aşağıdaki tanımlamalardan en çok hangisi sizin durumunuza uyuyor?

Lütfen sadece bir kutu işaretleyiniz.

☐ 1 Sigara içiyorum ve bırakmayı düşünmüyorum t2trans
☐ 2 Sigara içiyorum ve bırakmayı isteyip istemediğimi bilmiyorum
☐ 3 Sigara içiyorum ve bırakmayı istiyorum ama ne zaman bilmiyorum
☐ 4 Sigara içiyorum ve bırakmayı istiyorum ama gelecek 6 ay içinde değil
☐ 5 Sigara içiyorum ve gelecek 6 ay içinde bırakmayı istiyorum
☐ 6 Sigara içiyorum ve gelecek 30 gün içinde bırakmayı istiyorum

16. Sigarayı bırakmak için şu an kendinizi ne kadar hazır hissediyorsunuz?

Lütfen tahmininize en yakın sayıyı işaretleyiniz:

t2stoptermo

0 — 1— 2 — 3 — 4 — 5 — 6 — 7 — 8 — 9 — 10

0 = Bırakmak için hiç hazır değilim

10 = bırakmaya tamamen hazırım

17. Sigarayı bırakmayı istediğinizde çevrenizden daha fazla destek görmek ister misiniz?

☐ 2 Evet *t2supportw*

☐ 1 Hayır

Evetse, kimlerden? ______________________ *t2supportw_off*

18. Genel olarak sigara hakkında ne düşünüyorsunuz?
Aşağıdaki deyişlere ne kadar katılıyorsunuz?

Lütfen her satırda bir kutu işaretleyiniz. Doğru ya da yanlış cevap yoktur. Sadece düşüncelerinizi belirtiniz:

		**Tamamen Katılıyorum**	**Biraz Katılıyorum**	**Pek Katılmıyorum**	**Hiç Katılmıyorum**	
		1	2	3	4	
a)	Sigara içmek can sıkıntısına iyi gelir..	☐	☐	☐	☐	*t2ea*
b)	Sigara ardında kötü bir koku bırakır	☐	☐	☐	☐	*t2eb*
c)	Sigara içmek modern bir görünüş sağlar	☐	☐	☐	☐	*t2ec*
d)	Sigara içmek cildi daha çabuk yaşlandırır	☐	☐	☐	☐	*t2ed*
e)	Sigara insanı gevşetir ve rahatlatır.	☐	☐	☐	☐	*t2ee*
f)	Sigara içmek diğer insanların sağlığını da bozar	☐	☐	☐	☐	*t2ef*
g)	Sigaranın tadı güzeldir	☐	☐	☐	☐	*t2eg*
h)	Restorant, kahve ve bar gibi kamuya açık yerlerde sigara içilmesinin yasaklanması konusunda ne düşünüyorsunuz?	☐	☐	☐	☐	*t1eh*

19. Sigara içme isteğiniz var, fakat sigara içmek istemiyorsunuz: Sigara içmemek için, ne yapabilirsiniz? *(als OFFENE Frage stellen, TN müssen frei antworten >> Ablenkung vom Craving mit Gedanken, Mund und Händen)*

☐	*t2alt1*	Kitap/ gazete okurum	☐	*t2alt6*	Su içerim
☐	*t2alt2*	Yürüyüş, spor yaparım, hareket ederim	☐	*t2alt7*	Sigara içilen yerlerden uzak dururum
☐	*t2alt3*	Sakız çiğnerim	☐	*t2alt8*	Ev işleri yaparım (çamaşır/ temizlik)
☐	*t2alt4*	Televizyo, bilgisayar	☐	*t2alt9*	Uyurum, dinlenirim
☐	*t2alt5*	Meyve/ sebze yerim	☐	*t2alt10*	Başka şeyler: _________________

20. Sigaranın içinde blunan, sağlığa zarar veren 3 önemli maddeyi ve sağlığa nasıl zarar verdiğini belirtiniz (als OFFENE Frage stellen, TN müssen frei antworten)

		**Sigaranın içindeki 3 zararlı madde**			**Bu maddelerin sağlığa zararları**
☐	*t2inh1*	Nikotin	☐	*t2inh1a*	Bağımlılık yapar
☐	*t2inh2*	Katran	☐	*t2inh2a*	Kanser yapar, solunum yollarına ve akcigere zarar verir
☐	*t2inh3*	Karbonmonoksit	☐	*t2inh3a*	Kalp krizi, beyin kanaması, nefes darlığı
☐	*t2inh4*	Diğeri: ___________________	☐	*t2inh4a*	Diğeri: _________________________

Kursla ilgili sorular (bütün katılımcılar)

21. Sizinde katılığınız sigara kursunu organize eden dernek veya cami ile hala ilişkiniz var mı?

☐ 1 Hayır ☐ 2 Evet *t2verein*

Eğer cevabınız evetse, bu dernek ve camide sigara / sigara bırakma konuları güncelmi ve herhangi bir etkinlik düzenlendi mi?

☐ 1 Hayır konu güncel değil ve bu konuda herhangi bir etkinlik düzenlemedi *t2impact*
☐ 2 Bilmiyorum
☐ 3 Evet, sigara konusu üzerine öncekinden daha fazla konuşuluyor
☐ 4 Evet, hala sigarayı bırakma ile ilgilenen başka kişiler var
☐ 5 Evet, bu konuyla ilgili etkinlikler düzenleniyor-hangi etkinlik? ___________ *t2aktion*

22. Kurs, sigarayı bırakma denemenizde size yardımcı oldu mu?
Lütfen sadece bir kutu işaretleyiniz.

hilfver

- ☐ 1 Çok yardımcı oldu
- ☐ 2 Biraz yardımcı oldu
- ☐ 3 Şöyle böyle
- ☐ 4 Pek yardımcı olmadı
- ☐ 5 Hiç yardımcı olmadı

23. Sigarayı bırakma kursu size ve diğerlerine başka alalarda da (günlük konular, ilişki, grup içinde öğrenmek vb.) yardımcı oldu mu?
Lütfen sadece bir kutu işaretleyiniz.

hilfges

- ☐ 1 Çok yardımcı oldu
- ☐ 2 Biraz yardımcı oldu
- ☐ 3 Şöyle böyle
- ☐ 4 Pek yardımcı olmadı
- ☐ 5 Hiç yardımcı olmadı

24. Sigara Bırakma Kursunu çevrenizde ilgi duyan insanlara tavsiye eder misiniz?
Lütfen yalnızca bir kutu işaretleyiniz.

t2empfehl

- ☐ 1 Evet, kesinlikle ederim
- ☐ 2 Hehralde ederim
- ☐ 3 Bilmiyorum
- ☐ 4 Herhalde etmem
- ☐ 5 Hayır, kesinlikle etmem

25. Sigarayı bırakma kursunda, sizin sigarayı bırakmanıza daha çok yardımcı olacak başka neler yapılabilirdi? *t2offen1*

✎ ....

26. Kursun daha da iyileştirilmesi için başka önerileriniz var mı? *t2offen2*

✎ ....

Anketimizi doldurduğunuz için teşekkürler!
