# Supplementary material for: An adapted smoking-cessation intervention for Turkish-speaking migrants in Switzerland: Predictors of smoking outcomes at one-year follow-up
Source: PLoS One. 2021 Mar 18;16(3):e0247157. doi: 10.1371/journal.pone.0247157 (PMC7971503; doi:10.1371/journal.pone.0247157)
Supplement: S6 File — T2 questionnaire for smokers. (PDF) [file pone.0247157.s006.pdf]

Evaluation of smoking cessation courses for Turkish-speaking migrants in Switzerland

T2 questionnaire for smokers

DO NOT FILL IN! (to be filled in by the project team)

Course number|________| *course code*

Subject number: |__ __ __ __ __ __ __ __ ____ *pbnr*

Date of the last course attendance:
__ __ : __ __ : __ __ __ __ *t2ku_day2ku_mont2ku_yah*
Day Month Year

Date on which FB T2 was filled in
__ __ : __ __ : __ __ __ __ *t2fb_day2fb_mont2fb_yah*
Day Month Year

Entry questions

1. Did you take part in the entire stop smoking course without skipping a session?

☐ 2 Yes, I never missed a session and attended all of them *withoutabs*

☐ 1 No, I missed at least one session.

How many sessions did you miss? ______ Meetings *freqabs*

2. Have you stopped smoking in the last 4 months?

☐ 1 No *t2stop*

☐ 2 Yes, once

☐ 3 Yes, several times

➡ **If you have stopped smoking (but are smoking again) in the past 4 months, please→ continue with <u>question 3</u>**

➡ **If you have <u>not stopped smoking in the</u> past 4 months please continue →with <u>question 5</u>**

Questions about smoking cessation - only participants who have stopped smoking at least once (but are now smoking again)

3. What was your longest smoke-free time period (amount of consecutive days) in the last 4 months? (except for abstinence from smoking due to illness or hospitalization)?

| __ __ __ days **(if no longer known exactly,**
enter the **approximate number of days)** *permanent stop*

from: __ __ : __ __ : __ __ __ __ __ **(if no longer known exactly,** *betag, bemon, bejahr*
Day Month Year **enter approximate date)**

until: __ __ : __ __ : __ __ __ __ __ **(if no longer known exactly,** *end date, end month, end year*
Day Month Year **enter approximate date)**

4. In connection with the attempt to stop smoking, did you use drugs/preparations containing nicotine or did you use other methods of treatment?

Check one box in each line

	Applicable	Not applicable	
(a) nicotine skin patches	☐ 2	☐ 1	*t2hilfa*
(b) nicotine gum	☐ 2	☐ 1	*t2hilfb*
(c) Tablets containing nicotine for under the tongue	☐ 2	☐ 1	*t2hilfc*
(d) Drug "Zyban®".	☐ 2	☐ 1	*t2support*
(e) Drug "Champix	☐ 2	☐ 1	*t2help*
(f) Acupuncture	☐ 2	☐ 1	*t2hilff*
(g) hypnosis	☐ 2	☐ 1	*t2hilfg*
(h) Other: ________________________________	☐ 2	☐ 1	*t2hilfh* *t2help_off*
(i) Nicorette® Inhaler	☐ 2	☐ 1	*t2hilfi*
(j) Nicorette® Spray	☐ 2	☐ 1	*t2hilfj*
(k) smoke stop line	☐ 2	☐ 1	*t2help*

Questions on smoking behaviour (all participants)

5. How often do you smoke?
Please tick only one box.

- ☐ 1 Daily *t2-frequent*
- ☐ 2 Several times per week (___ times per week) *T2frequently2*
- ☐ 3 Once a week
- ☐ 4 Less frequently (___ times per month) *T2-frequently4*

6. How much do you generally smoke on a day that you smoke?

(a) ____ cigarettes *t2freqzigi*

(b)_____cigars *t2freqziga*

(c) ______Pipe *t2freq whistl*

(d) ______hookahs *t2freqw*

(e)_______ 'joints' (hashish / 'grass' / cannabis) *t2freqjoi*

(f) _______E-cigarettes *t2frequent*

7. How soon after waking do you smoke your first cigarette?
Please tick only one box.

- ☐ 4 Within 5 minutes *t2fag1*
- ☐ 3 Within 6 to 30 minutes
- ☐ 2 Within 31 to 60 minutes
- ☐ 1 After more than 60 minutes

8. Where and how often do you smoke at home?

Check one box in each line.

	frequently	rarely	never	
a) living room	☐ 2	☐ 1	☐ 0	*t2homea*
b) bedroom	☐ 2	☐ 1	☐ 0	*t2homeb*
c) children's room	☐ 2	☐ 1	☐ 0	*t2homec*
d) bath / Toilet	☐ 2	☐ 1	☐ 0	*t2homed*
e) kitchen	☐ 2	☐ 1	☐ 0	*t2homee*
f) balcony / garden / roof terrace	☐ 2	☐ 1	☐ 0	*t2homef*
g) Other room:______________________	☐ 2	☐ 1	☐ 0	*t2homeg* *t2homeg_off*

9. Smoking in a car: Do you or someone who lives with you own a car?

☐ 1 No

☐ 2 Yes

t2autoa

	frequently	rarely	never	
a) If so, how often do you or other passengers/drivers smoke in the car?	☐ 2	☐ 1	☐ 0	*t2autob*

10. How many people live with you in the same household? (including yourself)

Please enter the number: └────┘ Persons living in my household *t2mitbewo*

11. How many people who live with you smoke? (including yourself)

Please enter the number: └───┘ people smoke *t2withbewora*

12. How many of your 10 most important family members and friends in Switzerland smoke cigarettes?

Please enter the number:: └───┘ people smoke *t2people*

13. Has anyone close to you also tried to stop smoking because you tried to do so in the course?

☐ 1 No *t2other*
☐ 2 Yes

		Are these people still smoke-free today?	
If so, who?	1__________________	☐ 1 No 2☐ Yes	*t2p1 & t2rf1*
	2__________________	☐ 1 No 2☐ Yes	*t2p2 & t2rf2*
	3__________________	☐ 1 No 2☐ Yes	*t2p3 & t2rf3*
	4__________________	☐ 1 No 2☐ Yes	*t2p4 & t2rf4*

14. Why are you still smoking?

Please tick one box in each line.

		**Applies**	**Not applicable**	
		2	1	
a)	My friends/colleagues are still smoking	☐	☐	*grunda*
b)	My wife, husband, partner, is still smoking	☐	☐	*basically*
c)	I kept putting off quitting	☐	☐	*grundc*
d)	It is so hard to quit/ I still don't know how I will manage to stop smoking	☐	☐	*Basically*
e)	I don't want to stop	☐	☐	*reasons*
f)	Other reasons: __________________________	☐	☐	*grundfgrundf_off*

15. Which of the following statements best describes your current situation?

Please tick only one box.

☐ 1 I smoke and do not think about quitting *t2trans*

☐ 2 I smoke and don't know if I want to stop

☐ 3 I smoke and want to stop, but do not know when

☐ 4 I smoke and want to quit, but not in the next 6 months

☐ 5 I smoke and want to quit in the next 6 months

☐ 6 I smoke and want to stop in the next 30 days

16. How strong is your readiness to stop smoking at this time?

Please circle the number that corresponds to your estimation:

t2stoptermo

0 — 1— 2 — 3 — 4 — 5 — 6 — 7 — 8 — 9 — 10

0 = not ready at all 10 = I am very ready

17. If you were trying to quit smoking, would you need/want more support from other people?

☐ 2Yes *t2supportw*

☐ 1No

If so, from who? ________________________________ *t2supportw_off*

18. What is your general opinion of smoking? In your opinion, what is the most accurate?

Check one box in each line. There are no right and wrong answers. Just express your opinion.

		I completely agree	I somewhat agree	I somewhat disagree	I disagree completely	
a)	Smoking helps against boredom.	☐1	☐2	☐3	☐4	*t2ea*
b)	Smoking leaves an unpleasant smell.	☐1	☐2	☐3	☐4	*t2eb*
c)	Smoking is a behavioural trend.	☐1	☐2	☐3	☐4	*t2ec*
d)	Smoking makes the skin age faster.	☐1	☐2	☐3	☐4	*t2ed*
e)	Smoking calms and relaxes.	☐1	☐2	☐3	☐4	*t2ee*
f)	Smoking is harmful to other people's health.	☐1	☐2	☐3	☐4	*t2ef*
g)	Smoking tastes good.	☐1	☐2	☐3	☐4	*t2eg*
h)	What is your opinion on the general ban on smoking in public places, restaurants, cafés and bars?	☐1	☐2	☐3	☐4	*t2eh*

19. If you feel like smoking a cigarette, but do not want to smoke: What can you do instead, to avoid smoking? *(ask as OPEN question, person must answer freely > distraction from craving with thoughts, mouth and hands)*

☐	*t2alt1*	Read book/ newspaper	☐	*t2alt6*	Drink water
☐	*t2alt2*	Walking, sports, exercise	☐	*t2alt7*	Avoid places where people smoke
☐	*t2alt3*	Chew gum	☐	*t2alt8*	Household activities (washing/cleaning)
☐	*t2alt4*	TV, Internet	☐	*t2alt9*	Sleep, relax
☐	*t2alt5*	Eating fruit/ vegetables	☐	*t2alt10*	Other: ________________________

20. Please name the 3 most important ingredients of a cigarette and their effects on your Health *(ask as OPEN question, person must answer freely)*

		**Ingredient**			**health impact**
☐	*t2inh1*	Nicotine	☐	*t2inh1a*	Addiction
☐	*t2inh2*	Tar	☐	*t2inh2a*	Cancer, affects respiratory tract & lungs
☐	*t2inh3*	Carbon monoxide	☐	*t2inh3a*	heart attack, stroke, decreased oxygen transport
☐	*t2inh4*	Other: ___________________	☐	*t2inh4a*	Other: _________________________

Questions about the course (all participants)

21. Do you still have contact with the club/mosque that organised the stop smoking course at that time? *(do not ask questions in informal groups)*

☐ 1 No ☐ 2 Yes *t2club*

If YES, is the topic smoking/smoking cessation in your club or your Mosque still up to date and were any activities carried out in addition?

☐ 1 No, the topic is no longer relevant and there were no activities on it *t2impact*

☐ 2 I don't know.

☐ 3 Yes, there is still more talk about smoking than before

☐ 4 Yes, there are other people interested in being smoke-free

☐ 5 Yes, there have been activities related to this - Which ones? *t2action*

____

22. Was the stop smoking course helpful for you <u>in trying to quit smoking</u>?
Please tick only one box.

☐1 Very helpful *helpful*
☐2 Rather helpful
☐3 Neither nor
☐4 Rather not helpful
☐5 Not helpful at all

23. Was the stop smoking course helpful for you in <u>other areas</u> (support with everyday questions, contact, learning in the group etc.)?
Please tick only one box.

☐1 Very helpful *auxiliary*
☐2 Rather helpful
☐3 Neither nor
☐4 Rather not helpful
☐5 Not helpful at all

24. Would you recommend this stop smoking course to interested people from your environment?
Please tick only one box.

☐1 Yes, I'm sure *t2recommendation*
☐2 Probably yes
☐3 I don't know.
☐4 Probably not
☐5 No, certainly not

25. What could have been done differently in the stop smoking course that would would have helped better? *t2open*

✐ ....

....

26. Do you have any further suggestions for improvement of the course? *t2open*

✐ ....

....

....

Many thanks for your cooperation!
